# Comprehensive anatomic ontologies for lung development: A comparison of alveolar formation and maturation within mouse and human lung

**DOI:** 10.1186/s13326-019-0209-1

**Published:** 2019-10-24

**Authors:** Huaqin Pan, Gail H. Deutsch, Susan E. Wert, On behalf of the Ontology Subcommittee, Namasivayam Ambalavanan, Charles Ansong, Maryanne E. Ardini-Poleske, Jacqueline Bagwell, Cliburn Chan, Gail H. Deutsch, Charles Frevert, Davera Gabriel, James S. Hagood, Carol B. Hill, Jeanne Holden-Wiltse, Anil G. Jegga, Thomas J. Mariani, Anna Maria Masci, Huaqin Pan, Wei Shi, David Warburton, Susan E. Wert, Kathryn A, NHLBI Molecular Atlas of Lung Development Program Consortium

**Affiliations:** 1 BioInformatics, Research Computing Division, RTI International, 3020 Cornwallis Road, Research Triangle Park, Raleigh, North Carolina 27709 USA; 2 Department of Pathology, Seattle Children’s Hospital, University of Washington School of Medicine, 4800 Sand Point Way NE, Seattle, Washington 98105 USA; 3 Department of Pediatrics, Perinatal Institute, Section of Neonatology, Perinatal and Pulmonary Biology, Cincinnati Children’s Hospital Medical Center/Research Foundation, University of Cincinnati College of Medicine, 3333 Burnet Ave, MLC7029, Cincinnati, OH 45229 USA

**Keywords:** Alveolarization, Biomedical ontology, Data annotation, Database, Lung-specific cell types, Molecular anatomy, LungMAP, OWL, Single-cell analysis, Web-based atlas

## Abstract

**Background:**

Although the mouse is widely used to model human lung development, function, and disease, our understanding of the molecular mechanisms involved in alveolarization of the peripheral lung is incomplete. Recently, the Molecular Atlas of Lung Development Program (LungMAP) was funded by the National Heart, Lung, and Blood Institute to develop an integrated open access database (known as BREATH) to characterize the molecular and cellular anatomy of the developing lung. To support this effort, we designed detailed anatomic and cellular ontologies describing alveolar formation and maturation in both mouse and human lung.

**Description:**

While the general anatomic organization of the lung is similar for these two species, there are significant variations in the lung’s architectural organization, distribution of connective tissue, and cellular composition along the respiratory tract. Anatomic ontologies for both species were constructed as partonomic hierarchies and organized along the lung’s proximal-distal axis into respiratory, vascular, neural, and immunologic components. Terms for developmental and adult lung structures, tissues, and cells were included, providing comprehensive ontologies for application at varying levels of resolution. Using established scientific resources, multiple rounds of comparison were performed to identify common, analogous, and unique terms that describe the lungs of these two species. Existing biological and biomedical ontologies were examined and cross-referenced to facilitate integration at a later time, while additional terms were drawn from the scientific literature as needed. This comparative approach eliminated redundancy and inconsistent terminology, enabling us to differentiate true anatomic variations between mouse and human lungs. As a result, approximately 300 terms for fetal and postnatal lung structures, tissues, and cells were identified for each species.

**Conclusion:**

These ontologies standardize and expand current terminology for fetal and adult lungs, providing a qualitative framework for data annotation, retrieval, and integration across a wide variety of datasets in the BREATH database. To our knowledge, these are the first ontologies designed to include terminology specific for developmental structures in the lung, as well as to compare common anatomic features and variations between mouse and human lungs. These ontologies provide a unique resource for the LungMAP, as well as for the broader scientific community.

## Background

Ontologies are formal representations of knowledge used to handle big data sets and information retrieval. Ontologies consist of standardized vocabularies of terms for individual entities (or objects) that are associated with a specific domain or field of knowledge. Anatomy ontologies are designed to capture biological concepts and descriptions in a way that can be easily categorized and analyzed with computer technology. The most visible biological application today is the Gene Ontology project [1], which provides a controlled vocabulary for cross-species comparisons of genes and gene products that are associated with biological processes, molecular functions, and cellular components. In recent years, ontologies have become indispensable tools for various molecular anatomy and atlas projects, including GUDMAP, molecular anatomy of genitourinary tract development in the mouse [2, 3]; Brain Maps 4.0, neuroanatomy of the rat brain [4]; Xenbase, Xenopus anatomy and development [5]; and FaceBase, craniofacial anatomy, development and malformations in a variety of species [6]. Ontologies also extend our ability to access prior knowledge from other model organisms, using cross-referenced linkages to existing ontologies or databases. Together, these ontologies provide innovative tools for knowledge representation and modeling of biologic and developmental relationships, as well as cellular and molecular processes.

While extensive research has been published on the molecular regulation of early lung formation and branching morphogenesis of the conducting airways (reviewed in [7–10]), less is known about the molecular mechanisms regulating expansion and maturation of the alveolar parenchyma during the later stages of lung development (reviewed in [11–16]). This period of lung development is critical for the formation of the distal gas-exchange region of the lung, which is marked by the generation of millions of highly vascularized alveoli that are the lung’s primary gas-exchange units (reviewed in [17, 18]). This process, termed *alveolarization* (or *alveologenesis*), increases the surface area and diffusion capacity of the lung, which are required for efficient exchange of oxygen and carbon dioxide after birth. Disruption of this process has significant clinical relevance for managing neonatal lung disease related to prematurity, neonatal respiratory distress, and abnormal lung growth [19–21].

The mouse is an important animal model for investigating human lung development, function, and disease [22–26]. Although there are many anatomic, histologic, and developmental similarities between these two species, significant variations exist in the architectural organization, connective tissue elements, and cellular composition of their lungs [27–29]. Lung development in both species proceeds in an orderly fashion in response to molecular mechanisms that control the initial formation and subsequent proliferation, differentiation, growth and maturation of the lung (reviewed [7–10]). Development of the lung is divided into several stages that extend throughout the fetal and postnatal periods of life [17, 30]. These stages include the *embryonic*, *pseudoglandular*, *canalicular*, *saccular*, and *alveolar* stages, which describe the histologic changes observed during development of the lung [17, 30–35]. Vascular maturation of the alveolar capillary bed in both species takes place during the last stage of lung development and is coincident with alveolar septation [17, 36–38]. Although lung development is similar in all mammalian species, the relative timing and/or length of each developmental stage varies from one species to another [17, 39, 40]. While maturation of the peripheral alveoli is initiated prior to birth in the human lung [30, 34, 41, 42], similar histological changes in the mouse do not begin until after birth [17, 43]. In both species, ongoing formation of additional alveoli continues into young adulthood [36, 37, 41, 43, 44].

Recently, a cooperative research project, the Molecular Atlas of Lung Development Program (LungMAP), was initiated by the National Heart, Lung, and Blood Institute to characterize and compare the molecular anatomy of mouse and human lungs, focusing on the later stages of lung development and maturation [45, 46]. LungMAP is a consortium composed of four research centers, a mouse hub, a human tissue repository, a central database termed Bioinformatics REsource ATlas for the Healthy lung (BREATH), and a data-coordinating center with a public web site (www.lungmap.net) [45, 46]. The BREATH database is an integrated open-access database that contains multiple datasets generated by a variety of analytical approaches to detect temporal-spatial changes in the developing lung. These include changes in 1) mRNA and microRNA expression, using microarrays and mRNA sequencing; 2) epigenetic control of gene expression, based on DNA methylation patterns; 3) protein, lipid and metabolite expression, using mass spectrometry imaging; 4) protein and mRNA expression, using high-resolution immunofluorescence confocal microscopy and high-throughput in situ hybridization; and 5) structural features, using three-dimensional (3-D) imaging [47–51]. Annotation and retrieval of information from these diverse datasets require a standardized vocabulary to integrate the molecular data with anatomic, histologic, and cellular imaging, in order to identify functionally and/or anatomically defined cell types in the developing lung.

To support this effort, we developed a comprehensive, high-resolution ontology, incorporating terms for well-defined anatomic structures, tissues, and cells found in the late fetal and postnatal mouse lung. Likewise, a detailed anatomic ontology for the late fetal and postnatal human lung was constructed and then harmonized with the mouse ontology in order to compare normal developmental processes between the two species. To our knowledge, this is the first ontology to include terminology specific for developmental structures in the mouse and human lung, including pulmonary, vascular, neural and immunologic components critical for lung function. It is also the first time that specific cell types have been incorporated into an anatomic ontology for the lung.

## Content and construction

### Methods

The abstract version of these anatomic ontologies was constructed using Protégé^1^ version 5.0.0 [52, 53] (https://protege.stanford.edu/about.php) and Web Ontology Language (OWL 2). This approach supports integration with other biological and biomedical ontologies. Scientific content was informed by review of the published literature (peer-reviewed research, reviews, textbooks, atlases, and medical dictionaries), by the authors’ expertise in lung development, anatomy, histopathology, and cell biology, and by annotation requirements for the BREATH database. Terminology and definitions already in use were adopted from existing ontologies, including the Mouse Gross Anatomy and Development Ontology (EMAP) [54] (https://bioportal.bioontology.org/ontologies/EMAP); the Mouse Adult Gross Anatomy Ontology (MA) [55, 56] (http://bioportal.bioontology.org/ontologies/MA); the Foundational Model of Anatomy (FMA) [57] (http://bioportal.bioontology.org/ontologies/FMA); the Uber Anatomy Ontology (UBERON) [58] (http://bioportal.bioontology.org/ontologies/UBERON); and the Cell Ontology (CL) [59, 60] (http://bioportal.bioontology.org/ontologies/CL). Additional resources for terms and definitions included the National Cancer Institute Thesaurus (NCIT) (https://ncit.nci.nih.gov/ncitbrowser/) and National Institute of Health (NIH) Medical Subject Headings (MeSH) (https://www.nlm.nih.gov/mesh/). Definitions derived from existing ontologies and resources were often modified to reflect lung-specific knowledge and expertise. Synonyms commonly used in the literature and in other ontologies were included to improve query searching. Where additional terms were required (i.e., terms that could not be drawn from existing ontologies), the published literature was reviewed for the most widely accepted terms, synonyms, and definitions. Multiple revisions were performed to refine both existing and newly introduced terms, as well as term definitions and synonyms. Construction of these ontologies is open-ended, so that additional anatomic terms and newly defined or molecularly distinct cell types can be incorporated as needed for annotation and linkage at a later date.

### Design of the ontology framework for the mouse lung

**Fig. 1:**
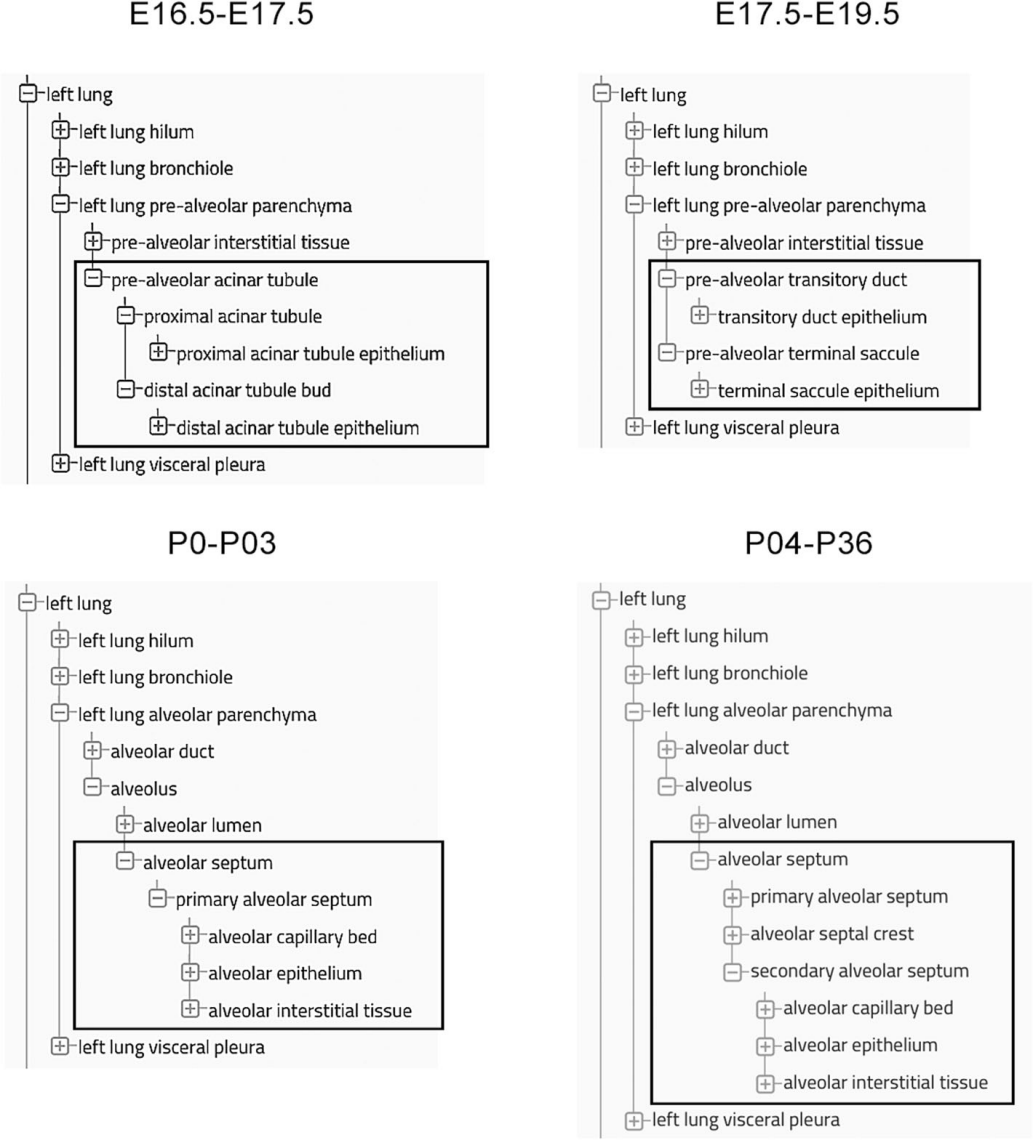
Terms associated with prenatal and postnatal development of the alveolar parenchyma organized by *age_range*. Development of the alveolar parenchyma is organized into 4 developmental stages, or *age_range(s)*, each with unique terminology for pre- and post-natal alveolar structures. These *age_range(s)* are E16.6-E17.5 (*canalicular stage*); E18.5-E19.5/birth (*saccular stage, prenatal period*); P0-P3 (*saccular stage, postnatal period*); P4-P36 (*alveolar stage*). Terms for the alveolar parenchyma (enclosed in boxes) differ with developmental stage as these structures evolve over time

A review of existing anatomic ontologies for the mouse, including UBERON, MA and EMAP, demonstrated that these ontologies had limited coverage of the fetal and postnatal mouse lung, especially for the later stages of lung development when alveolar growth, vascularization, septation, and maturation are initiated. In addition, developmental staging, specific terminology, and definitions for fetal lung structures and cells were often lacking in these established ontologies. As a result, we decided initially to organize the anatomic ontology for the mouse lung into four separate developmental time periods, or *age_range(s)* (Fig. 1), beginning with the *canalicular stage*, embryonic day (E) 16.5-E17.5, and ending with the *alveolar stage*, postnatal day (P) 4–36, of lung development. Since the intervening *saccular stage* (E17.5-P3) of lung development spans the perinatal period in the mouse, this stage is subdivided into two *age_range(s)*, i.e., a *prenatal (or early) saccular stage* (E17.5-E19.5) and a *postnatal (or late) saccular stage* (P0-P3) (Fig. 1). During these periods, terms associated with the formation and maturation of the alveolar parenchyma differ as this region evolves over time (enclosed boxes, Fig. 1).

Within each *age_range*, anatomic structures, tissues, and cells were organized into six major classes: trachea, bronchi, lung, vascular structures (including the pulmonary, bronchial, and lymphatic circulations), the autonomic nervous system, and the immune system (see https://www.lungmap.net/breath-ontology-browser/). As a rule, fetal lung development proceeds both spatially and temporally along the proximal-distal axis of the lung, so that formation, growth and differentiation of the proximal conducting airways, vasculature and nerves precede that of the distal alveolar parenchyma [61–66]. Therefore, each system was organized along the proximal-distal axis of the lung with increasing levels of granularity, i.e., from larger, lower resolution, extra-pulmonary structures (trachea, bronchi and pulmonary vessels) to smaller, higher resolution, intra-pulmonary structures (bronchioles, alveoli and alveolar capillaries), tissues, and cells. Microvascular structures of the alveolar-capillary bed were integrated into the alveolar parenchyma of the lung, since close apposition of the alveolar epithelium and adjacent capillary endothelium is critical for gas-exchange.

Considering the lung has over 40 different cell types that have been classified primarily by location, histology, function, and ultrastructural features [67–71], we integrated both general (e.g., epithelial, endothelial, interstitial cells) and specific (e.g., basal, ciliated, mucous, alveolar type II cells) cell types into the anatomic ontology. Recently, the availability of cell-specific markers, such as antibodies to transcription factors, intracellular proteins, and cell surface markers [7], has augmented our ability to detect and isolate lung-specific cell types and subpopulations, while advances in single-cell technology have identified molecularly distinct cells previously classified together as a single, specific, cell type [51, 72–75]. In order to improve data linkage and website queries based on single-cell data, a class termed *isolated lung cell types* was created for experimental cell types, i.e., newly defined cell types identified by single-cell RNA sequencing (scRNA-seq) analysis of isolated cells**.** The inclusion of general, specific, and isolated cell types is a major feature of this ontology that is not commonly found in many traditional anatomic and histologic ontologies.

### Construction of the mouse lung ontology

**Fig. 2:**
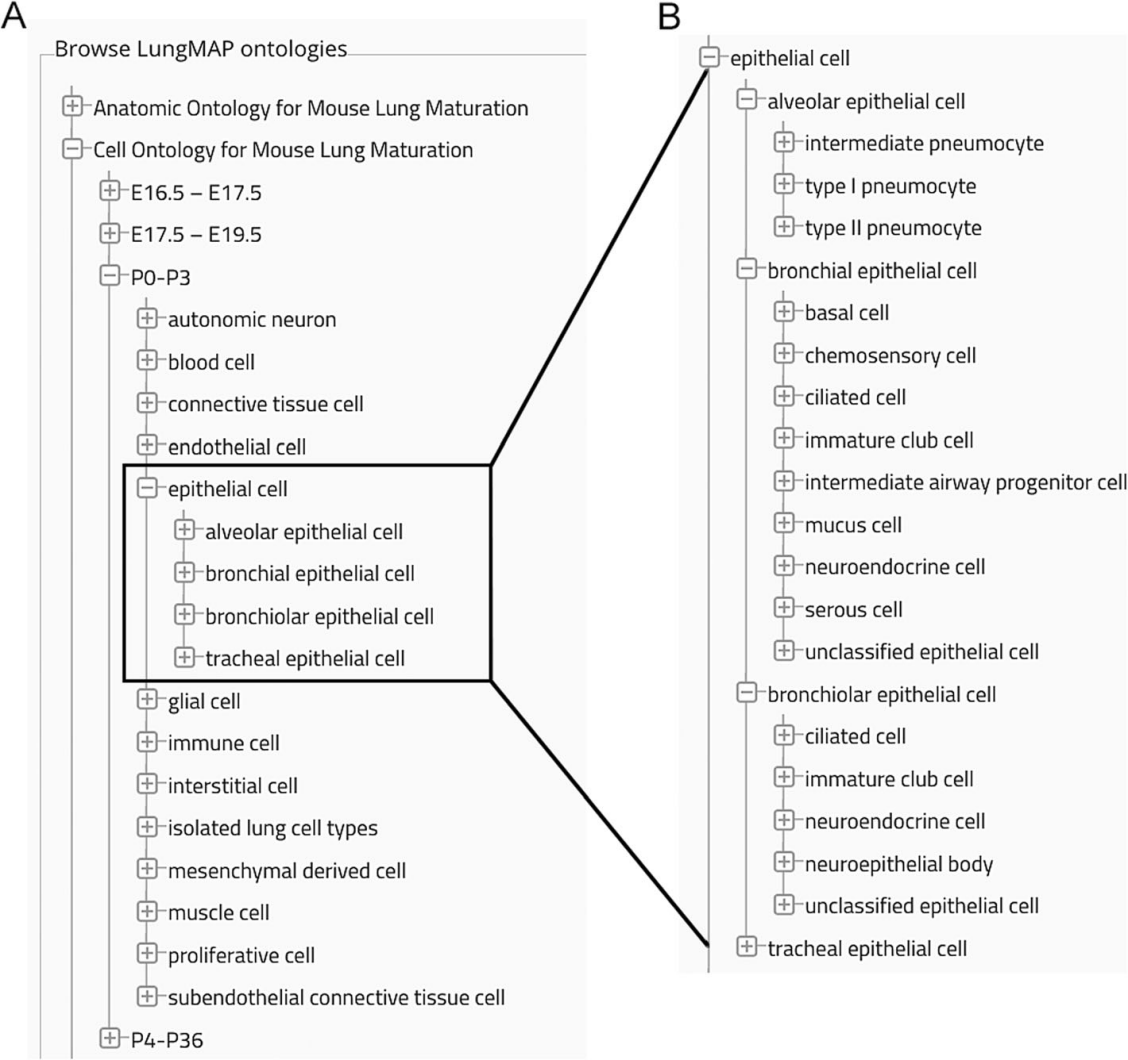
Cell ontology. General and specific cell types are organized into a separate tree of the ontology by developmental age (*age_range*) and then alphabetically. **a** There are 13 general cell classes for the mouse at P0-P3. The general cell type, *epithelial cell,* has four major subclasses: alveolar, bronchial, bronchiolar, and tracheal epithelial cells. **b** Each of these subclasses can be expanded into specific cell types, illustrating their distribution in the conducting airway and alveoli. A subclass termed *isolated lung cell types* was created for experimental cell types/subtypes identified by scRNA seq analysis

The anatomic ontology for the mouse lung is constructed as a partonomic (X is a part of Y, or *part_of*) hierarchy with separate trees for the different developmental stages or corresponding *age_range(s)* [3, 55, 76]. *Age_range* is an annotation property that is assigned to each term in the ontology. Each *age_range* is subdivided into separate respiratory, vascular, neural, and immunologic components that are organized along the proximal-distal axis of the lung**.** Each organ system, in turn, is populated with terms for well-characterized fetal, postnatal, and adult anatomic structures, distinct tissue compartments, and specific cell types. General and specific cell types incorporated into the anatomic ontology are also organized into a separate cell ontology, which is constructed using the *is_a* (subtype) relation. Within each *age_range*, cell types are listed alphabetically by class (major general cell types) and then by subclass (specific cell types) (Fig. 2). This strategy provides a comprehensive anatomic ontology that can be used at varying levels of resolution, i.e., with whole mounts, sectioned material or isolated tissues and cells.

**Fig. 3:**
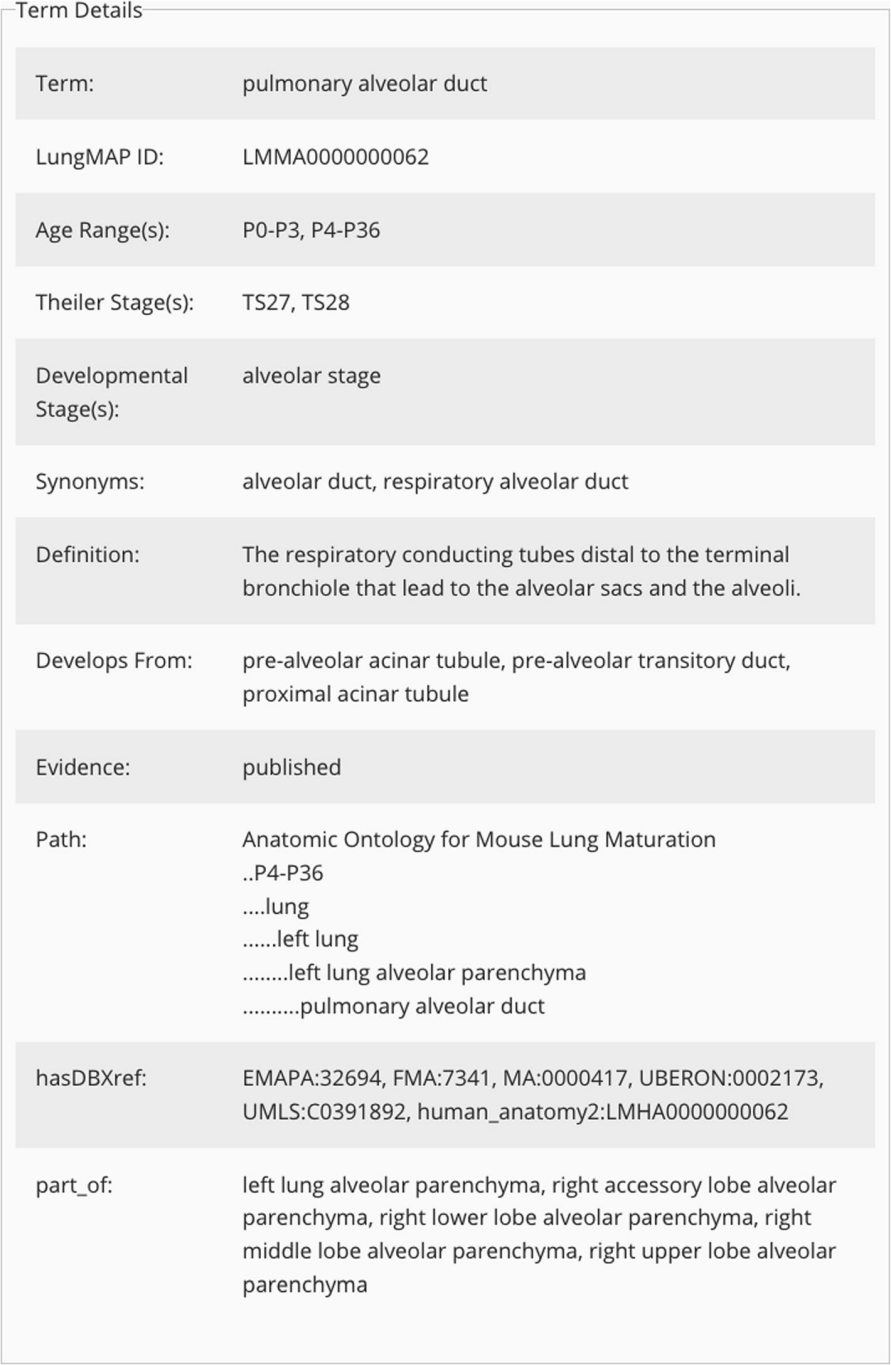
Term Details. Access to the Term Details box is achieved by selecting, or highlighting, any term in the Ontology Browser. The Term Details box contains the name of the term, a unique identifier (LungMAP ID), age range, Theiler stage, developmental stage, synonyms (if applicable), definition, developmental relationships (if known), evidence (experimental or published term), and ontology path, known database cross references (hasDBXref), and multiple parents

This ontology for the mouse contains 283 terms, including 167 for anatomic structures, 48 for tissues, and 68 for cells, which are distributed by developmental age and organ system (Table 1). It is displayed in the LungMAP’s website browser as a tree structure that can be expanded and collapsed as desired (see https://www.lungmap.net/breath-ontology-browser/). Access to term details is achieved by selecting or highlighting any term in the browser. This brings up the term detail box, which displays a unique identifier (i.e., a **L**ung**M**AP **M**ouse **A**natomy ID: LMMA_00XXX), a name (term or label), synonyms, and a definition for each individual entity (or object) (Fig. 3). This information, along with annotation properties and relations, are then incorporated into the formal ontology (Additional file 1**).** Annotation properties, i.e., specific attributes, features, characteristics or values that are associated with each individual object, are displayed in Table 2. The annotation property, *evidence*, was created to indicate terms that are either 1) well-established published terms or 2) experimental terms based on gene expression profiles generated by mRNA analysis of isolated cells or by scRNA-seq data. The special annotation property of *display_order* enabled proximal-distal organization of anatomic structures and tissues. *Relations*, or attributes describing how a class or an individual object relates to other classes and/or objects in the ontology [77], are displayed in Table 3. Although the primary relation used to construct this ontology is *part_of*, five additional relations are included to enrich the terms in the ontology**.** These included both spatial (*adjacent_to, continuous_with, branching_part_of*) and developmental (*develops_from*) relations, whose inclusion is designed to empower web-based queries related to complex molecular and cellular interactions.

**Table 1:** Distribution of terms for the mouse lung by anatomy, organ system, and developmental stage

Category	Number of terms
*Domain*	
Anatomy	167
Tissue	48
Cell	68
*Organ system*	
Trachea	40
Bronchus	44
Lung	94
Vascular system	74
Autonomic nervous system	20
Immune system	41
*Developmental stage (age_range)* ^*a*^	
Canalicular Stage (E16.5-E17.5)	231
Prenatal (early-mid) Saccular Stage (E17.5-E19.5)	229
Postnatal (mid-late) Saccular Stage (P0-P03)	253
Alveolar Stage (P04-P36)	262

^a^, includes terms for all organ systems at each developmental stage

**Table 2:** Annotation properties for the fetal and postnatal mouse lung

Annotation property	Examples
*Age_range*	E16.5-E17.5, E17.5-E19.5, P0-P3, P4-P36
*Developmental_Stage*	Canalicular Stage (E16.5-E17.5) Prenatal Saccular Stage (E17.5-E19.5) Postnatal Saccular Stage (P0-P03) Alveolar Stage (P04-P36)
*Theiler_Stage*	TS24 (16 dpc), TS25 (17 dpc), TS26 (18 dpc) TS27 (P0-P3), TS28 (P4-Adult)
*Evidence*	Published Experimental
*Has_abbreviation*	Abbreviations
*Has_synonym*	Alternative names

**Table 3:** Ontology class relations for both mouse and human lung

Relations	Examples
*part_of*	“left lung bronchiole” is *part_of* “left lung”
*is_a*	alveolar epithelium *is_a* epithelium “lymphatic endothelial cell” *is a* “endothelial cell”
*adjacent_to*	“basement membrane” is *adjacent_to* “alveolar epithelium” “pericyte” is *adjacent_to* “alveolar capillary endothelium”
*branching_part_of*	“lateral bronchiole” is a *branching_part_of* the “central bronchiole” “terminal bronchiole” is a branching_part_of the “lateral bronchiole” “alveolar duct” is a branching part of “the terminal bronchiole”
*develops_from*	“alveolus” *develops_from* “pre-alveolar terminal saccule”, which *develops_from* “pre-alveolar acinar tubule” “secondary alveolar septum” *develops_from* “alveolar septal crest”, which *develops_from* “primary alveolar septum”
*continuous_with*	“trachea” is *continuous_with* “bronchus”, which is *continuous_with* “central bronchiole”

Existing terms from relevant ontologies were adopted where applicable. Terms were classified as *existing* if they could be mapped to current ontologies and/or to additional vocabularies (e.g., NCIT or NIH MeSH) found in the National Center of Biomedical Ontology (NCBO) Bioportal (https://www.bioontology.org) [78, 79]. In general, terms drawn from other ontologies describe well-known anatomic structures, tissues and cells, but rarely include a complete description of lung-specific tissues and cells. Where additional terms were required, the scientific literature was reviewed for the most widely accepted terms, synonyms, and definitions. Additional terms most often included tissue structures and cells specific for the lung, such as *alveolar lumen*, *alveolar capillary bed*, and *lipofibroblast*, or for lung-specific developmental structures and cells, such as *acinar tubule*, *alveolar septal crest*, and *immature club cell*. Many of these developmental terms have been used for years in the literature to describe the histology of the developing lung [30, 34] and were incorporated into the ontologies, where possible, due to their long and extensive use in the literature. As a result, there are 159 (56%) terms cross-referenced to existing ontologies and 124 (44%) additional terms drawn from the literature for the mouse anatomic ontology (Table 4). This represents a significant expansion of current anatomic ontologies for the developing and adult mouse lung.

**Table 4:** Distribution of terms for the mouse lung drawn from existing ontologies and resources

Category	NoNo.	%	Ontology	No.
Anatomic ontologies	15,144	51%	Uber Anatomy Ontology (UBERON)	68
			Foundational Model of Anatomy (FMA)	34
			Cell Ontology (CL)	38
			Human Developmental Anatomy Ontology, abstract version 2 (EHDAA2)	4
Non-anatomic ontologies	15	5%	National Cancer Institute Thesaurus (NCIT)	4
			NIH Medical Subject Headings (MeSH)	3
			BRENDA Tissue and Enzyme Source Ontology (BTO)	5
			Systematized Nomenclature of Medicine, Clinical Terms (SNOMED CT)	1
			Neuroscience Information Framework, Standard Ontology (NIFSTD)	1
			Systemized Nomenclature of Medicine, International Version (SNMI)	1
All existing terms	159	56%		159
Additional terms^a^	124	44%		124
Total	283	100%		283

^a^, Terms drawn from the scientific literature but not found in any of the ontologies listed in the NCBO Bioportal

### Development of the human lung ontology

As for the mouse, review of existing human anatomic and developmental ontologies, such as FMA, UBERON, and the Human Developmental Anatomy Ontology (http://bioportal.bioontology.org/ontologies/EHDAA2) [80], revealed limited coverage of fetal and postnatal human lung structures, especially those associated with the late stages of lung development when alveolar growth, vascularization, septation and maturation are initiated. In the mouse, the alveolar stage of lung development begins postnatally around P4 and is complete by P36 [32, 36]. In contrast, this stage of human lung development begins prior to birth, at approximately 36 weeks of gestational age (GA), and continues after birth into the first few years of life [41]. The human lung ontology was patterned initially on the *alveolar stage* of mouse lung development and then revised to reflect the unique differences in architectural organization, anatomic structures, tissue components, and cellular composition between the two species. Commonly used synonyms were included to improve harmonization search capabilities across the two species. As was done for the mouse, general and specific cell types were incorporated in to the anatomic ontology, and an additional cell ontology was constructed in which the cells were listed alphabetically by class (major general cell type) and then by subclass (specific cell type) using the primary relation, *is_a*. Annotation properties (Table 2) and class relations (Table 3) were harmonized with those developed for the mouse lung ontology.

### Comparison of mouse and human lung anatomy

Although the general anatomic organization of the mature human and mouse lung is similar, there are significant variations in the gross architecture, as well as in the distribution of connective tissue elements and in cellular composition along the airways (Additional file 2). These variations are due primarily to differences in size between the two species and partly to differences in the shape of the lungs. These differences are described below and are reflected in the corresponding ontologies, where anatomic structures are captured as specific classes of objects whose anatomic features are related through the hierarchical, partonomic design of these ontologies.

#### Lung Lobes and Segmental Bronchi

Both the human and mouse lung are composed of multiple lobes that vary in number and organization between the two species. The human lung has 2 lobes on the left and 3 on the right, while the mouse lung has 1 lobe on the left and 4 on the right. In the human, there are multiple, intrapulmonary, *segmental bronchi* containing cartilage and submucosal glands [44], while in the mouse, the cartilaginous airways end with the lobar bronchi (Additional file 2, Fig. 4). Each segmental bronchus in the human lung supplies a distinct bronchopulmonary segment, which is subdivided into multiple pulmonary lobules. While the human lung has extensive interlobular and segmental connective tissue dividing each lobe into individual lobules or segments, the mouse lung is not subdivided into these smaller units (Additional file 2).

**Fig. 4:**
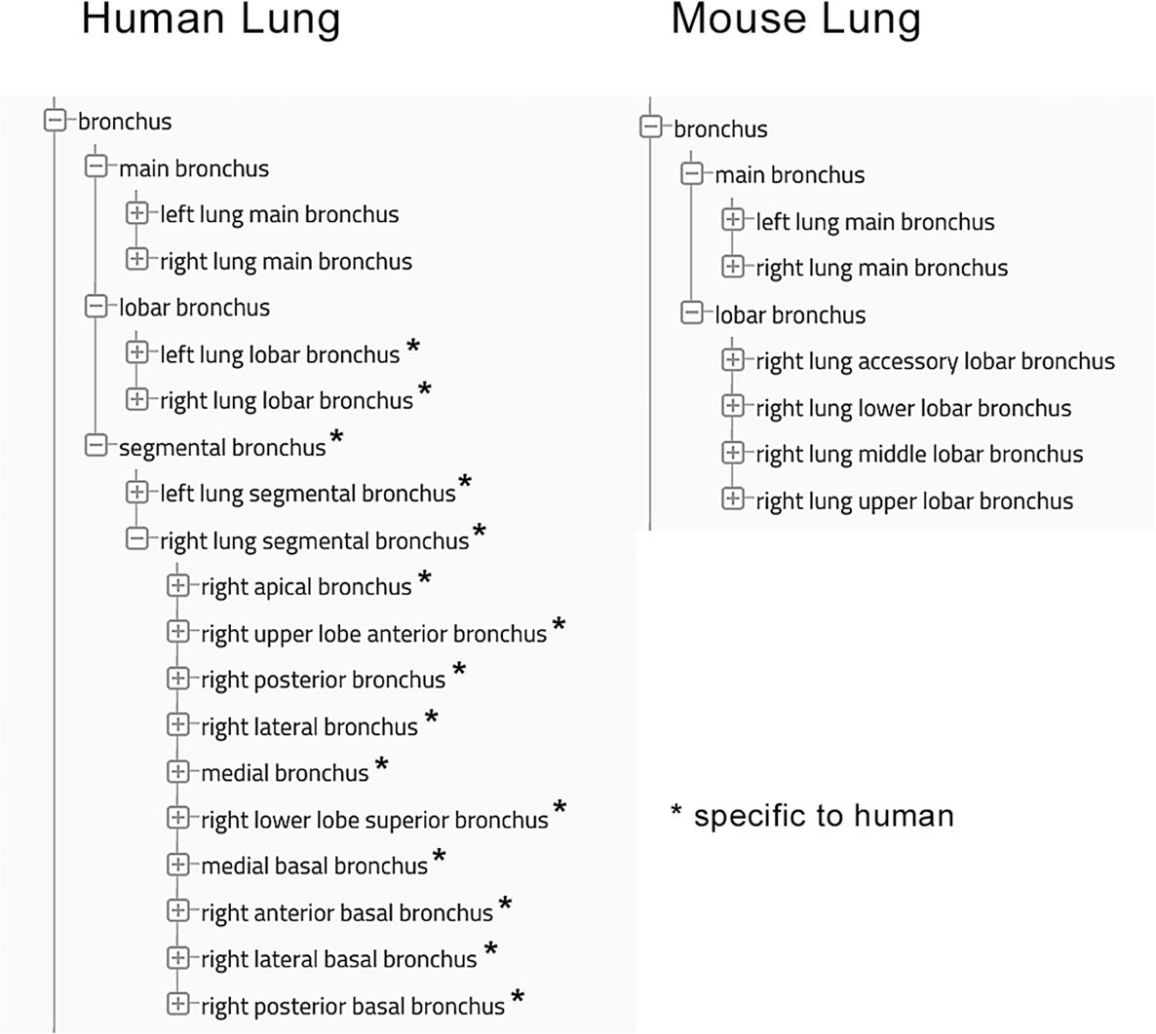
Comparison of the proximal airways in human and mouse lung. Differences in the nomenclature of the human and mouse proximal conducting airways are captured in the anatomic ontologies. The human lung has additional intrapulmonary segmental (tertiary) bronchi. *Structures specific to the human lung

#### Conducting Airways and Branching Patterns

**Fig. 5:**
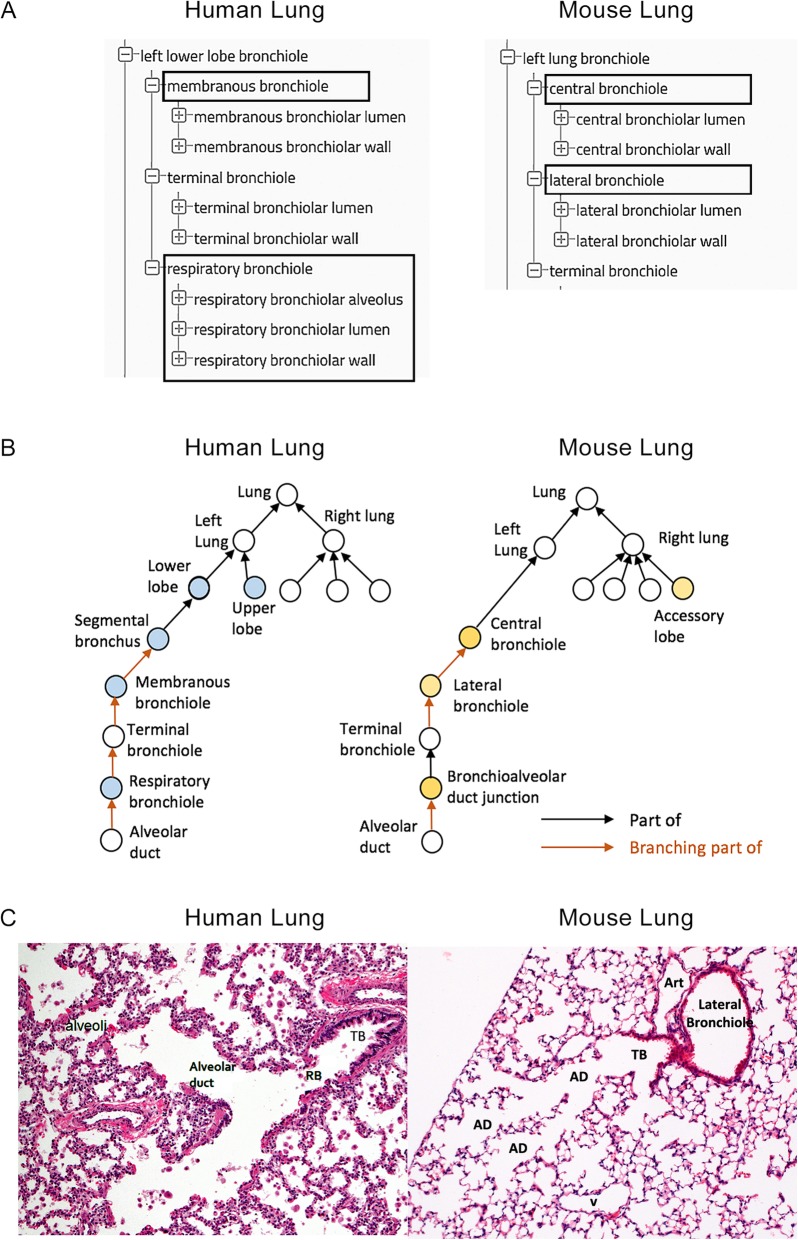
Comparison of the conducting airways between human and mouse. **a** Organization of the bronchiolar structures in the anatomic ontology with differences enclosed in boxes. **b** Schematic drawing of the conducting airways in human and mouse. The circles without color indicate similar structural organization and terminology. Those with color indicate differences in structural organization. Differences between human and mouse with respect to the relationships *part_of* and *branching_part_of* are highlighted with black and orange, respectively, reflecting differences in size and complexity of the human and mouse lung. **c** Histologic comparison of the bronchioles in human and mouse lung stained with hematoxylin and eosin; original magnification, 10X; Art, pulmonary artery; TB, terminal bronchiole; RB, respiratory bronchiole; AD, alveolar duct

The conducting airway of the lung is a tree-like structure that is formed during development by repetitive branching of the bronchial tubules into the surrounding mesenchyme. The segmental bronchi in the human lung exhibit an irregular pattern of dichotomous (formed by repeated bifurcations) branching which gives rise to ~ 16–23 generations of branches from the trachea to the gas exchange region of the lung [81–83]. Airway branching in the mouse is more asymmetric and gives rise to ~ 13–17 generations of airways [84]. In the mouse, there is a single axial or central airway (*central bronchiole*) that runs the length of each lobe with multiple lateral branches (*lateral bronchioles*) that form along its length. Each of these lateral branches bifurcate 3 to 4 times before ending in the *terminal bronchioles* [85, 86]. In the human lung, the terminal bronchioles branch into 2 to 3 generations of alveolarized *respiratory bronchioles***.** These bronchioles subsequently branch into multiple *alveolar ducts* that are lined entirely by *alveoli*. In contrast, there are no respiratory bronchioles in mice. Instead there are short terminal bronchioles with an abrupt transition to thin-walled alveolar ducts at the *bronchoalveolar duct junction***.** These differences are captured in the ontologies for mouse and human lungs, as shown in Fig. 5.

#### Cellular Composition

There are also important differences between the human and mouse lung with respect to the cellular composition of the airway epithelia, which varies along the proximal-distal axis [28, 87–90]. These differences are reflected in the construction of the anatomic and cell ontologies for both species. In both species, the trachea and proximal conducting airways are lined by pseudostratified columnar epithelium, while the more peripheral conducting airways are lined by cuboidal epithelium. In the human lung, the more proximal, intrapulmonary, cartilaginous airways (bronchi) resemble that of the trachea and are lined by tall, pseudostratified, columnar epithelia composed of *basal, ciliated*, *club*, *serous*, *mucus*, *intermediate* and *neuroendocrine cells*, and exhibit abundant *submucosal glands*. In contrast, the more proximal intrapulmonary conducting airways (bronchioles) of the mouse are lined by low columnar epithelia, composed primarily of ciliated and club cells with clusters of neuroendocrine cells largely located at airway branch points. There are no basal cells and only rare mucous cells. In the human lung, the respiratory bronchioles are lined by cuboidal epithelia, alternating with thin-walled alveoli lined by squamous *alveolar type I pneumocytes*. In the mouse, the terminal bronchioles are lined by cuboidal epithelial cells with an abrupt transition (bronchioalveoar duct junction) to the *alveolar duct*, which is lined by squamous type I pneumocytes. In general, the relative proportion of these different cell types varies along the proximal-distal axis in both human and mouse airways [28, 89, 90]. While the variation in cell types between human and mouse airways are captured in these ontologies, the relative proportions of these cells along the conducting airway are not.

### Comparison and classification of terms

Overall, we generated 283 and 301 classes for the mouse (Table 4) and human (Table 5) lung, respectively, with 224 common terms, 12 analogous terms, 65 terms unique to human, and 47 terms unique to mouse between the two ontologies (Additional file 3). Repeated rounds of comparison and harmonization were performed to validate and refine the classification of these terms into common, analogous, and unique terms. The process we used to compare and classify terms for the human and mouse ontologies is described below (Additional file 4).

- *Common terms* describe identical structures and/or cells found in both human and mouse lung, e.g., *bronchial cartilaginous ring*, *primary alveolar septum*, and *type II pneumocyte(s).*
- *Analogous terms* describe structures that are anatomically similar in both human and mouse lung, e.g., the *membranous bronchiole* in the human lung is analogous to the *central bronchiole* and *lateral bronchiole* in the mouse lung.
- *Unique terms* describe structures and/or cells that are present in one species, but not in the other. Typically, these terms represent tissue structures that are less developed in the mouse compared to the human lung, primarily due to differences in size. For example, the *bronchiolar adventitia* and *bronchiolar lamina propria* are well-developed in the human lung and can be distinguished by histologic examination. On the other hand, these two structures are more difficult to distinguish in the mouse lung and thus have been combined into one broader term, e.g., *bronchiolar connective tissue*. These terms also include terms for species-specific structures or cells that are present in only one or the other species, but not in both, such as 1) the *inferior lingular bronchus* and *respiratory bronchiole*, found in the human lung but not in the mouse, and 2) the *right lung accessory lobe*, *venous cardiac muscle*, *cardiomyocyte*, and *chemosensory cell*, which are found in the mouse, but not in the human lung.

**Table 5:** Distribution of terms for the human lung drawn from existing ontologies and resources

Category	No.	%	Ontology	No.
Anatomic ontologies	167	55%	Uber Anatomy Ontology (UBERON)	75
			Foundational Model of Anatomy (FMA)	47
			Cell Ontology (CL)	41
			Human Developmental Anatomy Ontology, abstract version 2 (EHDAA2)	4
Non-anatomic ontologies	22	7%	National Cancer Institute Thesaurus (NCIT)	5
			NIH Medical Subject Headings (MeSH)	5
			BRENDA Tissue and Enzyme Source Ontology (BTO)	7
			Systematized Nomenclature of Medicine, Clinical Terms (SNOMED CT)	3
			Radiology Lexicon (RADLEX)	2
All Existing Terms	189	63%		189
Additional Terms^a^	112	37%		112
Total	301	100%		301

^a^, Terms drawn from the scientific literature, but not found in any of the ontologies listed in the NCBO Bioportal

### Harmonization of terms

When harmonizing terms for anatomic concepts between the human and mouse ontologies, several decisions based on LungMAP objectives and current knowledge of the variations in human and mouse lung anatomy were considered. In general, we decided to adopt the UBERON and MA naming convention to be consistent. We used these terms as the primary term for a given structure and then used alternate terms from the literature as synonyms for annotation. For example, *right lung lower lobe* was used as the primary term for this lobe, while *right caudal lobe* and *right diaphragmatic lobe* were used as synonyms.

We found, however, that certain terms were used for both general (mouse) and specific (human) anatomic structures, which could cause confusion. The term *respiratory*, for example, is used in our anatomic ontologies to designate a specific structure in the human lung, the *respiratory bronchiole*, while in the MA, it is used as a general term to describe the intrapulmonary system of bronchioles in the mouse lung. In the human anatomic ontology, we use *respiratory bronchiole* to indicate that this structure is both a conducting airway and a gas-exchange structure with alveoli, i.e., a hybrid structure found in the human lung, but not in the mouse lung [91]. In the mouse anatomic ontology, we assigned specific terms to the individual components of the intrapulmonary system of bronchioles, i.e., *central, lateral*, and *terminal bronchioles*, instead of using the more general term.

### Harmonization of terms with existing ontologies

During development of these ontologies, we adopted knowledge from relevant ontologies, adopting existing terms and definitions where applicable (Additional file 3). We searched for each term in the NCBO BioPortal and found that only 56% (159) and 63% (189) of the terms for the mouse and human anatomic ontology, respectively, could be mapped to existing ontologies (Tables 4 and 5). The following criteria were used for mapping and harmonizing our ontology terms with those found in existing ontologies.

- Terms with names identical to those found in existing anatomic or cell ontologies, such as *bronchial epithelium* (UBERON_0002031) and *pulmonary nerve plexus* (UBERON_0002009, were adopted for use in our ontologies,
- For terms in our ontologies with similar, but not identical names associated with existing anatomic or cellular ontologies, we had to decide if these terms represented the same structure or concept in both species. If so, we harmonized our original terms by changing them to those found in the existing ontologies and then added our terms as synonyms. Several of the original names were terms commonly used in our LungMAP Research Centers. Therefore, including them as synonyms enables our users to find these terms in the LungMAP ontologies. For example, we changed *alveolar septum* to *interalveolar septum* (UBERON_0004893), *lymphoid macrophage* to *lymph node macrophage* (CL_0000868), and *right anterior basal bronchus* to *right anterior basal segmental bronchus* (FMA7418), and then added our original terms as synonyms.
- Terms with names found in existing BioPortal vocabularies or lexicons, but not in existing ontologies, were also included in our anatomic and cell ontologies. For example, *tracheal submucosa* (SNOMEDCT/4419000) and *telocyte* (MeSH/ D000067170) were not found in any existing ontology.

As a consequence, 44% (124) of the anatomic ontology terms for the mouse lung (Table 4) and 37% (112) of those for the human lung (Table 5) were not found in the BioPortal. These additional terms, such as *alveolar capillary bed* and *pulmonary arteriole*, were added to both ontologies.

## Utility and discussion

### Data annotation and retrieval

These ontologies provide a controlled vocabulary of well-defined terms that describe the developing and adult lung. These terms are used to tag and retrieve posted and/or stored data when searching or navigating the LungMAP website, which facilitates data annotation, retrieval, and linkage related to the BREATH database. The inclusion of synonyms and other alternate terms allows users, who may not be lung specialists, to access relevant data describing developmental concepts, functional relationships, and molecular interactions between the tissues and cells of the lung. The inclusion of annotation properties and relations is designed to enable more advanced user queries. For example, one potential application is to identify all datasets annotated with a specific structure (e.g., *alveolus*) and then query for 1) spatially related structures (*adjacent_to)*; 2) developmentally related structures *(develops_from);* or 3) developmental stage (*alveolar stage*).

**Fig. 6:**
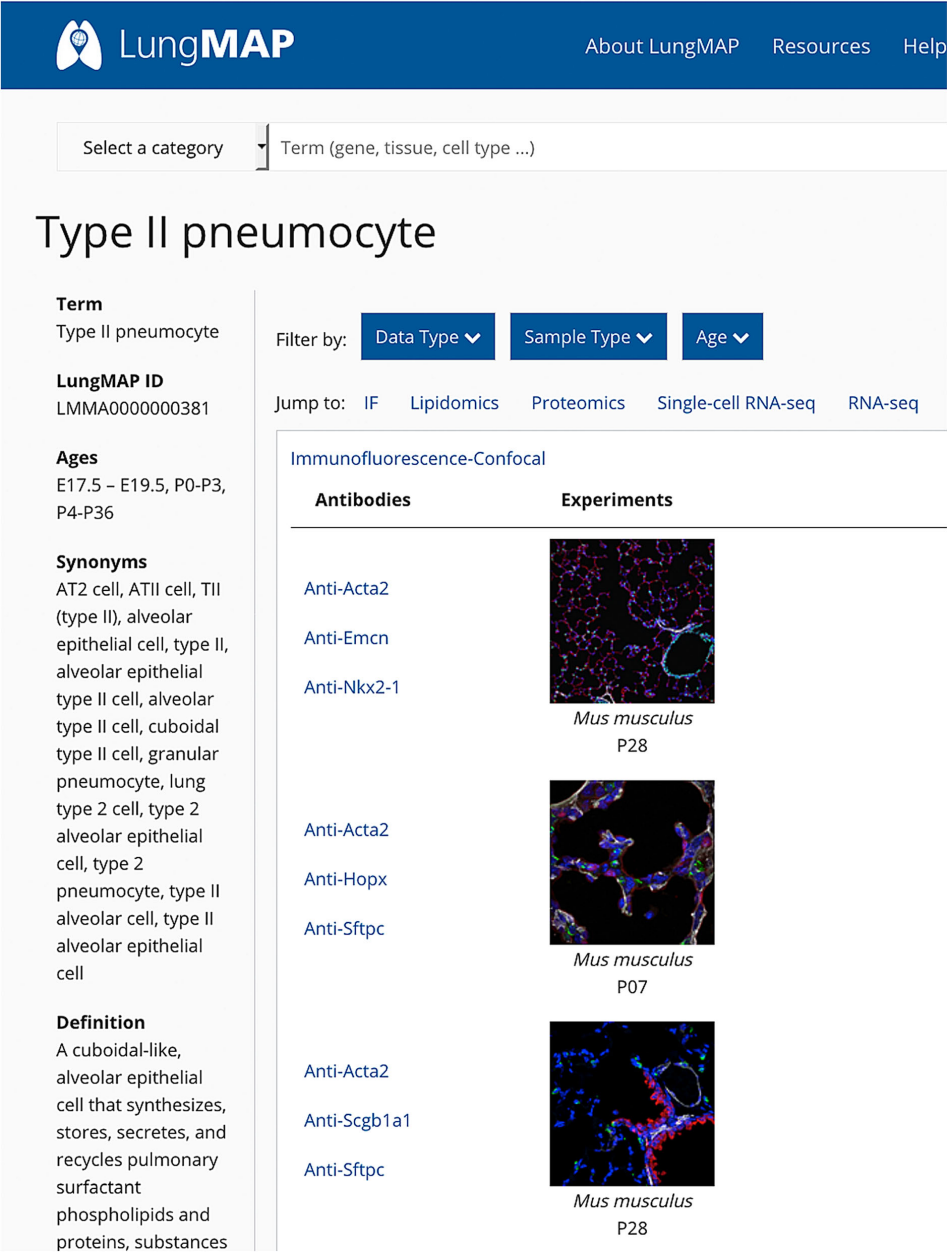
Utility of ontology terms for data retrieval and linkage. A keyword search using the term, “type II pneumocyte” returns a summary page that displays term details, as well as links to datasets for five different datatypes annotated with this term in the mouse: immunofluorescence-confocal, lipidomics, proteomics, scRNA-seq, RNA-seq. The search can be narrowed by data type, sample type, and age of sample. Shown is a partial list of imaging experiments for the postnatal mouse lung

**Fig. 7:**
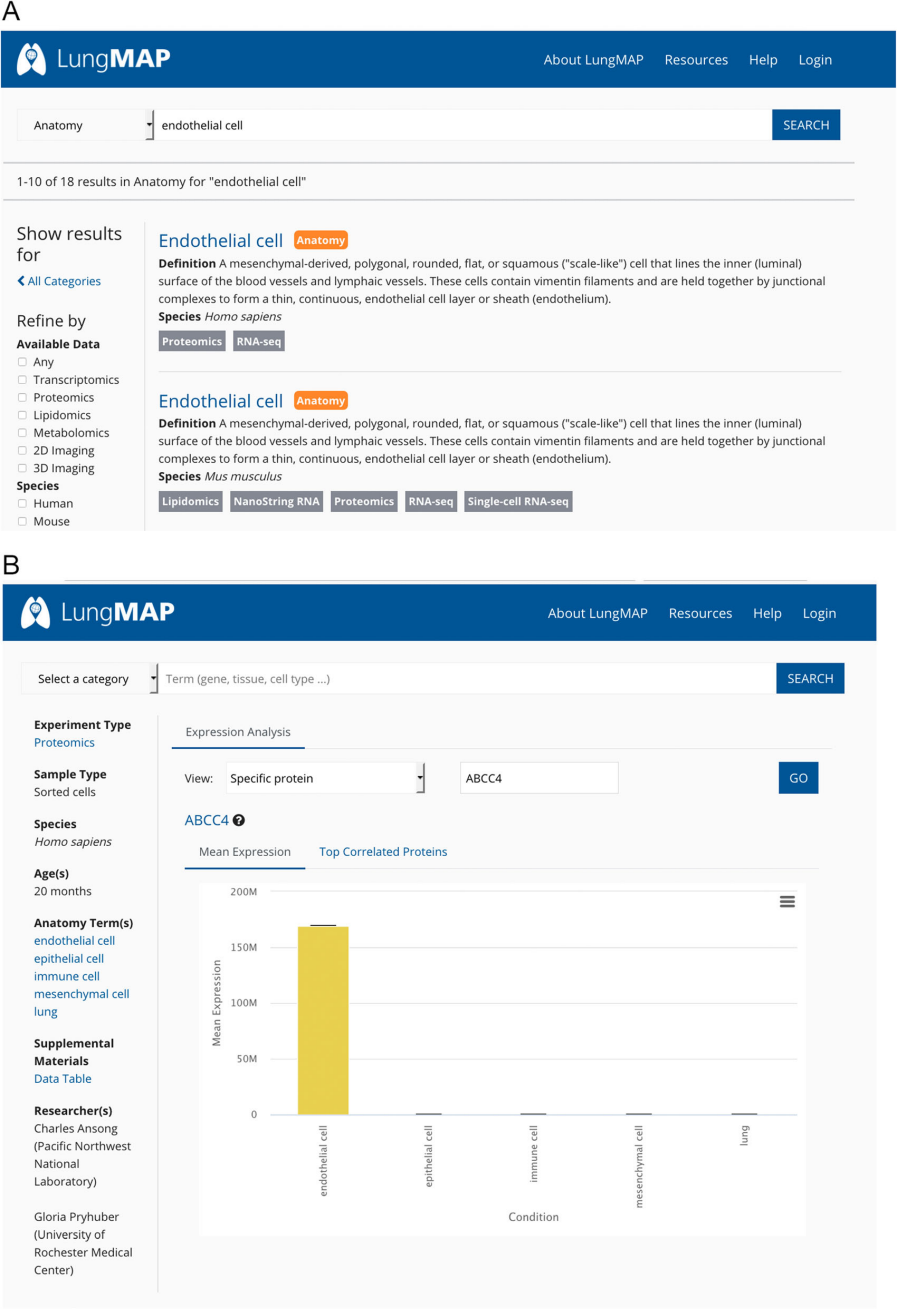
Utility of ontology terms for retrieval of datasets comparing isolated human and mouse cells. **a** A search for “endothelial cell” returns a results page with links to two different data types for isolated human endothelial cells and five for isolated mouse endothelial cells. **b** Selecting “Proteomics” for “*Homo sapiens*” returns a list of proteins found in isolated human lung cells, including endothelial, epithelial, immune, and mesenchymal cells. Shown are protein expression levels for the ATP-binding cassette C4 (ABCC4) transmembrane protein, also known as multidrug resistance-associated protein 4, which is highly expressed in endothelial cells compared to other cell types

All experimental data are annotated with species and age of the sample(s) harvested for the experiment, as well as with relevant anatomy terms and/or cell types. Linkage to experimental data annotated with a selected anatomic feature or cell type is achieved by entering the term in the search box on the website’s home page (https://www.lungmap.net). For example, a keyword search using one of these terms, *type II pneumocyte*, links the user to a summary page that provides term details, as well as links to multiple datasets annotated with this term for the mouse (Fig. 6), including immunofluorescence-confocal (IF), lipidomics, proteomics, scRNA-seq, and RNA-seq data types. Likewise, a search for more general cell types, such as *endothelial cell*, returns a results page with links to four different data types for isolated mouse cells and two for isolated human cells (Fig. 7a). Selecting “Proteomics” for “*Homo sapiens*” returns a list of proteins found in isolated human lung endothelial cells (Fig. 7b). Thus, the application of these comparative ontologies for annotation purposes facilitates the direct linkage and display of human and mouse data at the website using common, analogous, or unique terms for the two species.

### Image annotation

**Fig. 8:**
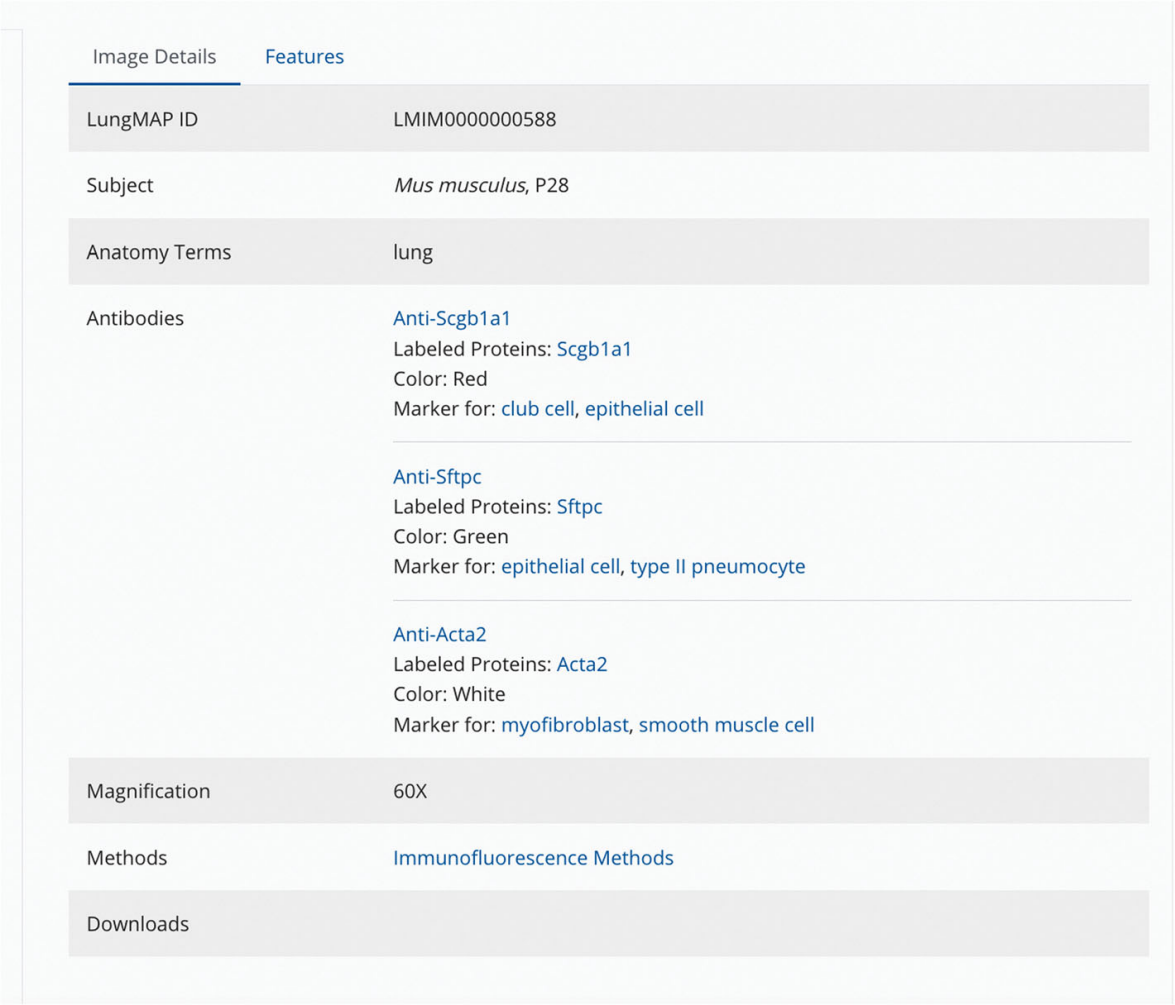
Image Details box for annotated immunofluorescence confocal images. Each immunofluorescence image has an Image Details box with basic information about the image (image ID, sample information, magnification), immunoassay details (antibodies, labeled proteins, cell-specific marker information, detection method) and interpretation (image notes) with links (highlighted in blue) to datasets in the BREATH database annotated with these terms

**Fig. 9:**
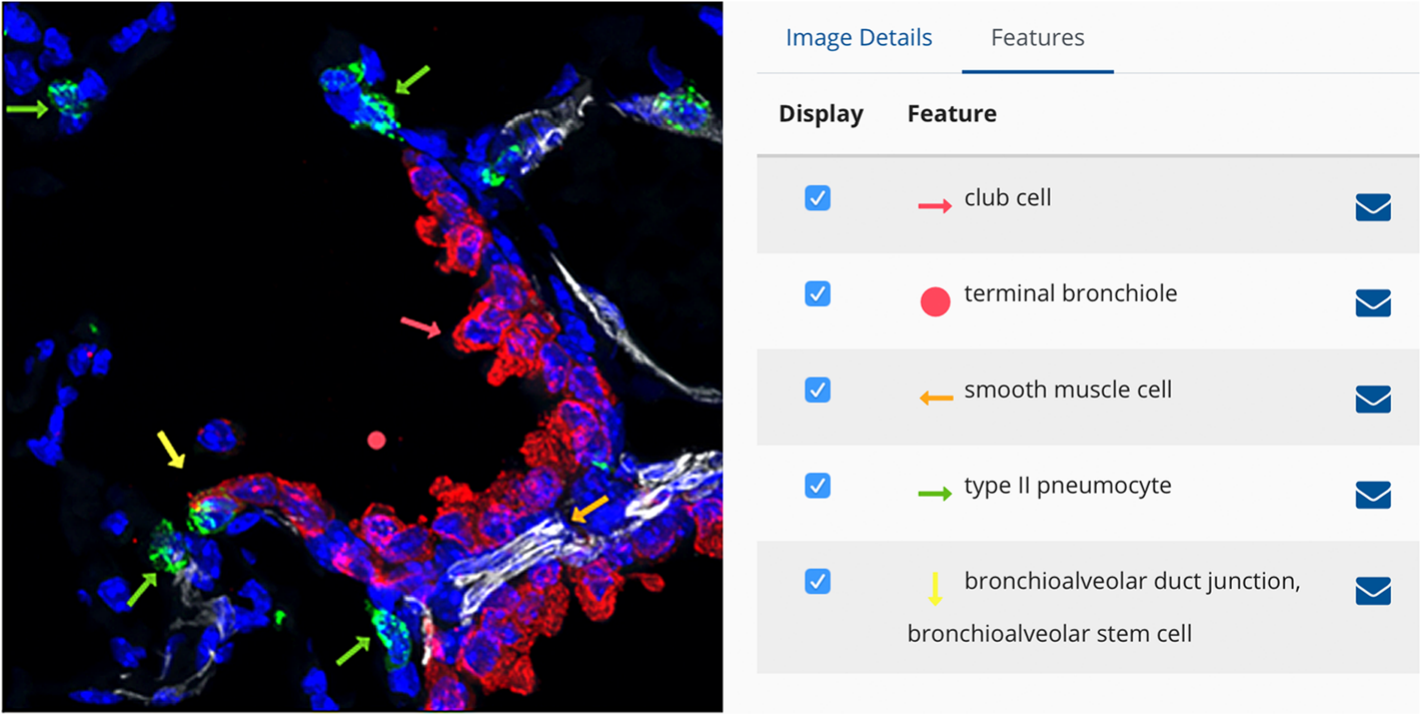
Annotated immunofluorescence confocal image demonstrating use of ontology terms. This is a triple-labeled image of a P28 (alveolar stage) postnatal mouse lung stained with antibodies to alpha-smooth muscle actin, ACTA2 (white), a club cell marker, SCGB1A1 (red), and an alveolar type II pneumocyte marker, SFTPC (green). Nuclei are labeled with DAPI. Annotation terms (right panel) are drawn from the ontology. Original magnification, 60X. Original image provided by J. A. Kitzmiller and J. A. Whitsett and can be found at the CCHMC LGEA web portal, https://research.cchmc.org/lungimage/ [92, 93]

Currently, this ontology is used to annotate anatomic features and cells on a variety of images, including 1) histologic features on images stained with hematoxylin and eosin, 2) gene expression patterns on digoxigenin-stained in situ hybridization images, 3) cell-specific expression patterns of proteins on immunofluorescence images captured by confocal microscopy, and 4) illuminated metabolites imaged using nano-DESI mass spectrometry. Images posted to the LungMAP website are annotated in two ways: 1) a nascent effort at machine annotation by algorithms based on training sets and 2) a manual image annotation tool designed for this purpose (https://www.lungmap.net/resources/annotation/) [21, 22]. Each annotated image has an *Image Details* panel (Fig. 8) that provides basic information about the image, including image ID, magnification, notes and links to the original image file and detailed experimental methods for download. Sample information (species, age, tissue), as well as antibody, target protein and cell-specific marker information are also found here. Links are provided to 1) additional antibody and target protein information, 2) term details for the cell types associated with cell-specific markers, and 3) BREATH data sets annotated with these terms. The *Features* panel includes annotation information for the tissues and cells found in the image. Manual annotation of tissues and cells found in each image **i**s achieved by searching for the desired ontology term and then applying it to the image using one of the available symbols (Fig. 9). This generates a list of terms that correspond to the highlighted or labeled structures (called “features”) seen on the image. Image annotation requires familiarity with the lung and is performed currently by knowledgeable annotators and curators within the LungMAP consortium. Through the annotated images, visitors to the website can learn quickly about anatomic and histologic structures, cell-specific markers, and protein and/or gene expression patterns in the developing and/or mature mouse and human lung.

### Molecular anatomy of tissues and cells

**Fig. 10:**
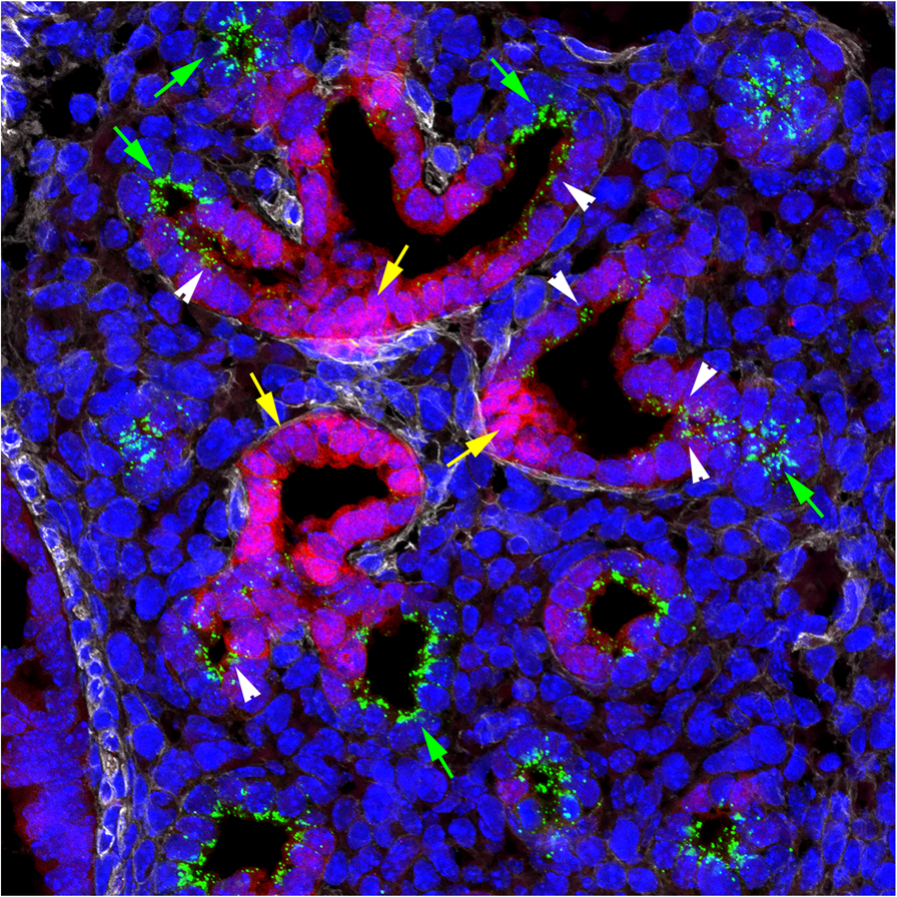
Immunofluorescence analysis of SFTPC and HOPX expression in the E16.5 mouse lung. Triple-labeled section of an E16.5, C57BL/6, fetal mouse lung stained for HOPX (red), SFTPC (green), ACTA2 (white), and counterstained with DAPI (blue) to visualize the nuclei. HOPX (intense bright red/magenta cytoplasmic staining) is expressed in epithelial cells of the proximal acinar tubules (yellow arrows), while SFTPC (multiple bright green punctate staining) is expressed in epithelial cells of the distal acinar tubules/buds (green arrows). Between the proximal and distal regions of the acinar tubules, there is a transition zone (white arrowheads) with less intense HOPX staining and fewer SFTPC puncta in the cytoplasm of the epithelial cells. Original magnification = 60X. Original image provided by J. A. Kitzmiller and J. A. Whitsett and can be found at the CCHMC LGEA web portal, https://research.cchmc.org/lungimage/ [92, 93]

Due to the complexity of the developing lung, current ontologies are not always sufficient to describe gene and/or protein expression patterns at high resolution with respect to localization in specific anatomic compartments, tissues, and cells. To address this gap, we constructed a comprehensive anatomic ontology focused to the molecular analysis of tissues and cells during alveolarization. A unique feature of this ontology is the inclusion of general, specific, and isolated cell types. This feature supports the annotation of scRNA-seq data derived from isolated cells, as well as the identification and localization of newly defined cell types based on molecular profiling [73, 74, 92–95]. For example, it has been suggested that there is a common alveolar progenitor cell for type I and type II pneumocytes in the fetal lung, which co-expresses cell-specific markers for these two cell types [74, 96–98]. These cell-specific markers include surfactant protein C (SFTPC) for type II pneumocytes and Hop Homeobox (HOPX), Advanced Glycosylation End Product-Specific Receptor (AGER) and Podoplanin (PDNP) for type I pneumocytes. Analysis of scRNA-seq data for cells isolated at E16.5 and E18.5 demonstrated subpopulations of alveolar epithelial cells expressing both SFTPC and HOPX [98]. Co-expression of HOPX and SFTPC was observed by immunofluorescence in a subset of epithelial cells as early as E15.5 [98] and at E16.5 (Fig. 10). These dual-positive cells were located in a transition zone connecting the proximal (HOPX only) and distal (SFTPC only) acinar tubules (Fig. 10). In the anatomic ontology, these cells are described as *intermediate pneumocytes* (syn: *bipotent pneumocyte*) that are defined as epithelial cells with characteristics of both type I and type II precursor cells as determined by co-expression of HOPX and SFTPC, respectively. Immunolocalization of these cell-specific markers identified a unique anatomic and cellular location for these HOPX/SFTPC dual-positive cells, as well as a distinct molecular sub-compartment that was not readily visible at the histologic level without the use of these markers and immunofluorescence confocal microscopy (Fig. 10).

### Increased resolution

The development of these ontologies, which were built on existing anatomic ontologies, adds increased resolution to the terminology available for the human and mouse lung when compared to other ontologies for the lung. As described above, 56% (mouse) and 63% (human) of the terms in our combined ontologies were drawn from existing ontologies in the NCBO Bioportal, while 44% (mouse) and 37% human) additional terms were drawn from the scientific literature. These additional terms represent:

- Tissues found in compartments and locations specific for the lung, such as the *alveolar capillary bed*, *alveolar septal crest*, *pulmonary arteriole*, and *bronchial vein tunica adventitia.*
- Cells specific for the lung, such as the *alveolar interstitial cell* and *lipofibroblast*.
- Various landmarks, transition points, or anatomic spaces specific for the lung, such as the *right lower lobe hilum*, *bronchoalveolar duct junction*, and *alveolar lumen*.

This increased resolution enriches and expands existing human and mouse anatomic ontologies in multiple ways by 1) incorporating existing terms from non-anatomic ontologies into a well-defined anatomic ontology with hierarchical organizations and relationships and 2) incorporating additional terms that describe anatomic structures, tissues, and cells for specific regions and locations in the lung. The increased resolution of these ontologies enables precise mapping of molecular data to specific anatomic locations, tissue structures, and cells in the lung.

### Expansion of existing ontologies

These ontologies are the only anatomic and cell ontologies dedicated to descriptions of the developing and adult lung. Although these were constructed, in part, by adopting terms from existing ontologies, such as UBERON, CL, EMAP and MA**,** additional terms were drawn from the literature and incorporated into these ontologies**,** supplementing the terminology in current anatomic ontologies for mouse and human (Additional file 3). These ontologies expand current anatomic ontologies by the inclusion of terms specific for developmental structures in the lung. Additional terms are stage-specific and are organized into discrete developmental time periods, or *age_range(s),* which correlate with previously described stages of lung development, not currently represented in existing ontologies. Increased granularity, critical for the localization of new cell markers (i.e., RNA and proteins), is accomplished by the incorporation of specific cell types into their corresponding tissue compartments. Although tissue compartments and cells are not typically included in classic anatomic ontologies, these additions support the research goal of the LungMAP, i.e., to create a molecular, temporal-spatial map of the developing lung.

### Future development

These human and mouse anatomic lung ontologies will be maintained and updated on the LungMAP website as needed. Since construction is open-ended, these ontologies can be enhanced by incorporation of additional classes, terms, synonyms, annotation properties, and relations as required by future experimental data sets. Earlier developmental time points for the human lung (<GA36) will be added and harmonized with the mouse ontology as more donor tissues are acquired by the LungMAP’s human tissue core (https://www.lungmap.net/about/lungmap-team/human-tissue-core/). Newly defined and/or molecularly distinct cell types can be incorporated as needed, providing additional opportunities for the integration of anatomic and molecular data critical for the determination of cell fate and lineage relationships, cell-cell interactions and cell functions in the lung.

Currently, these ontologies are limited to qualitative features of the developing lung. They do not take into consideration quantitative traits, such as the number and geometric properties of the branching airways, the number of alveoli, or alveolar surface area critical for physiological function. Studies using computed tomography and 3-D imaging of the mouse and human lung are underway, however, which will require the addition of terms to support these studies. Anatomic landmarks and features (points, borders, surfaces and spaces), as well as spatial relationships relevant to both 2-D and 3-D images, will be added as lung whole mount and micro-CT images are acquired. At that time, a limited numbering system may be developed for the branching airways and for distinct pulmonary acini, visible by 3-D imaging. Finally, development of ontology applications may be required for new web-based tools designed for comparative visualization and analysis of human and mouse datasets at the LungMAP/BREATH website.

## Conclusion

Here we describe detailed ontologies that incorporate terms for anatomic structures, tissues, and cells involved in alveolar development and maturation within the mouse and human lung. These terms represent both commonly used and more specific terms for fetal and postnatal lung structures, tissues, and cells, which are incorporated into an interactive, searchable, web-based atlas, providing a common vocabulary for the annotation and integration of experimental datasets posted to the LungMAP’s BREATH database. These ontologies supplement and significantly expand current ontologies, which lack structural and cellular specificity, as well as the species divergence required for comparative anatomy of the lung. Synchronous development of these ontologies eliminates redundant and inconsistent terminology, enabling differentiation of true anatomic variations between the mouse and human lung. Identification and harmonization of common, analogous, and unique terminology for human and mouse lung enables comparative data linkage and molecular analyses between the two species, serving as a unique resource for the LungMAP and the broader research community.

## Supplementary information

**Additional file 1. **OWL file for the class *left lung alveolar parenchyma* with its annotation properties and relationships.

**Additional file 2..** Specific differences in lung structure between human and mouse.

**Additional file 3..** Comparison of ontology terms for human and mouse lung anatomy.

**Additional file 4..** Harmonization process comparing anatomic structures and terms in human and mouse ontologies.

## Data Availability

Access to the ontology browser is through the LungMAP website, https://www.lungmap.net/breath-ontology-browser/. Individual WORD, PDF, and OWL files can be found on the ontology home page at https://www.lungmap.net/resources/ontologies/. These ontologies are freely available without constraint at the NCBO BioPortal, as •Anatomic Ontology for Mouse Lung Maturation (LUNGMAP-MOUSE), available athttps://bioportal.bioontology.org/ontologies/LUNGMAP-MOUSE •Cell Ontology for Mouse Lung Maturation (LUNGMAP-M-CELL), available athttps://bioportal.bioontology.org/ontologies/LUNGMAP_M_CELL •Anatomic Ontology for Human Lung Maturation, https://bioportal.bioontology.org/ontologies/LUNGMAP-HUMAN •Cell Ontology for Human Lung Maturation, https://bioportal.bioontology.org/ontologies/LUNGMAP_H_CELL

## Abbreviations

3-D: 3-dimensional
BREATH: Bioinformatics REsource ATlas for the Healthy lung
CL: Cell Ontology
DNA: DeoxyriboNucleic Acid
EMAP: Mouse Gross Anatomy and Development Ontology
FMA: Foundational Model of Anatomy
GUDMAP: GenitoUrinary Development Molecular Anatomy Project
LungMAP: Molecular Atlas of Lung Development Program
MA: Mouse Adult Gross Anatomy Ontology
miRNA: MicroRNA
mRNA: Messenger RiboNucleic Acid
OWL: Web Ontology Language version
UBERON: Uber Anatomy Ontology Institute Thesaurus
NCIT: National Cancer Institute Thesaurus
NIH: National Institute of Health
MeSH: NIH Medical Subject Heading
E: Embryonic day
P: Postnatal day
scRNA-seq: Single-cell RNA sequencing
LMMA ID: LungMAP Mouse Anatomy IDentification number
NCBO: National Center for Biomedical Ontologies
GA: Gestational age
SNOMEDCT: Systematized Nomenclature of Medicine Clinical Terms
nanoDESI: Nanospray Desorption ElectroSpray Ionization
RNA-seq: RNA sequencing
SFTPC: Surfactant Protein C
HOPX: Hop Homeobox
AGER: Advanced Glycosylation End Product-Specific Receptor
PDPN: Podoplanin
syn: Synonym
2-D: 2-dimensional
micro-CT: Micro-computed tomography
*Art*: *Pulmonary artery*
*TB*: *Terminal bronchiole*
*RB*: *Respiratory bronchiole*
*AD*: *Alveolar duct*
ABCC4: ATP-binding cassette C4
ACTA2: Actin, alpha-2
*SCGB1A1*: Secretoglobin, family 1A, member 1
DAPI: 4′,6-diamidino-2-phenylindole
CCHMC: Cincinnati Children’s Hospital Medical Center
LGEA: Lung Gene Expression Analysis
RTI: Research Triangle Institute
HDAA2: Human Developmental Anatomy Ontology, abstract version 2
BTO: BRENDA Tissue and Enzyme Source Ontology
NIFSTD: Neuroscience Information Framework (NIF) Standard Ontology
SNMI: Systematized Nomenclature of Medicine, International Version
RADFLEX: Radiology Lexicon

## References

[1] Ashburner M, Ball CA, Blake JA, Botstein D, Butler H, Cherry JM (2000). Gene ontology: tool for the unification of biology. The Gene Ontology Consortium. Nat Genet.

[2] Georgas KM, Armstrong J, Keast JR, Larkins CE, McHugh KM, Southard-Smith EM (2015). An illustrated anatomical ontology of the developing mouse lower urogenital tract. Development.

[3] Little MH, Brennan J, Georgas K, Davies JA, Davidson DR, Baldock RA (2007). A high-resolution anatomical ontology of the developing murine genitourinary tract. Gene Expr Patterns.

[4] Swanson LW (2018). Brain maps 4.0-structure of the rat brain: an open access atlas with global nervous system nomenclature ontology and flatmaps. J Comp Neurol.

[5] Segerdell E, Ponferrada VG, James-Zorn C, Burns KA, Fortriede JD, Dahdul WM (2013). Enhanced XAO: the ontology of Xenopus anatomy and development underpins more accurate annotation of gene expression and queries on Xenbase. J Biomed Semantics.

[6] Brinkley JF, Borromeo C, Clarkson M, Cox TC, Cunningham MJ, Detwiler LT (2013). The ontology of craniofacial development and malformation for translational craniofacial research. Am J Med Genet C Semin Med Genet.

[7] Herriges M, Morrisey EE (2014). Lung development: orchestrating the generation and regeneration of a complex organ. Development.

[8] Kimura J, Deutsch GH (2007). Key mechanisms of early lung development. Pediatr Dev Pathol.

[9] Maeda Y, Dave V, Whitsett JA (2007). Transcriptional control of lung morphogenesis. Physiol Rev.

[10] Warburton D, El-Hashash A, Carraro G, Tiozzo C, Sala F, Rogers O (2010). Lung organogenesis. Curr Top Dev Biol.

[11] Chao CM, Moiseenko A, Zimmer KP, Bellusci S (2016). Alveologenesis: key cellular players and fibroblast growth factor 10 signaling. Mol Cell Pediatr.

[12] Hines EA, Sun X (2014). Tissue crosstalk in lung development. J Cell Biochem.

[13] McCulley D, Wienhold M, Sun X (2015). The pulmonary mesenchyme directs lung development. Curr Opin Genet Dev.

[14] Morrisey EE, Cardoso WV, Lane RH, Rabinovitch M, Abman SH, Ai X (2013). Molecular determinants of lung development. Ann Am Thorac Soc.

[15] Olave N, Lal CV, Halloran B, Pandit K, Cuna AC, Faye-Petersen OM (2016). Regulation of alveolar septation by microRNA-489. Am J Physiol Lung Cell Mol Physiol.

[16] Surate Solaligue DE, Rodriguez-Castillo JA, Ahlbrecht K, Morty RE (2017). Recent advances in our understanding of the mechanisms of late lung development and bronchopulmonary dysplasia. Am J Physiol Lung Cell Mol Physiol..

[17] Schittny JC (2017). Development of the lung. Cell Tissue Res.

[18] Wert SE, Polin RA, Abman SH, Benitz WE, Rowitch DH, Fox WW (2017). Normal and abnormal structural development of the lung. Fetal and neonatal physiology.

[19] Alvira CM (2016). Aberrant Pulmonary Vascular Growth and Remodeling in Bronchopulmonary Dysplasia. Front Med (Lausanne).

[20] Silva DM, Nardiello C, Pozarska A, Morty RE (2015). Recent advances in the mechanisms of lung alveolarization and the pathogenesis of bronchopulmonary dysplasia. Am J Physiol Lung Cell Mol Physiol.

[21] Lal CV, Bhandari V, Ambalavanan N, Pammi M, Lal CV, Wagner BD (2018). Genomics, microbiomics, proteomics, and metabolomics in bronchopulmonary dysplasia airway microbiome and development of bronchopulmonary dysplasia in preterm infants: A systematic review. Semin Perinatol.

[22] Baguma-Nibasheka M, Gugic D, Saraga-Babic M, Kablar B (2012). Role of skeletal muscle in lung development. Histol Histopathol.

[23] Nardiello C, Mizikova I, Morty RE (2017). Looking ahead: where to next for animal models of bronchopulmonary dysplasia?. Cell Tissue Res.

[24] Shapiro SD (2007). Transgenic and gene-targeted mice as models for chronic obstructive pulmonary disease. Eur Respir J.

[25] Tashiro J, Rubio GA, Limper AH, Williams K, Elliot SJ, Ninou I (2017). Exploring Animal Models That Resemble Idiopathic Pulmonary Fibrosis. Front Med (Lausanne).

[26] Wirsdorfer F, Jendrossek V (2017). Modeling DNA damage-induced pneumopathy in mice: insight from danger signaling cascades. Radiat Oncol.

[27] Rock JR, Randell SH, Hogan BL (2010). Airway basal stem cells: a perspective on their roles in epithelial homeostasis and remodeling. Dis Model Mech.

[28] Suarez C, Dintzis S, Frevert C, Treuting PM (2012). Respiratory. Comparative anatomy and histology: A mouse and human atlas.

[29] Tyler WS (1983). Comparative subgross anatomy of lungs. Pleuras, interlobular septa, and distal airways. Am Rev Respir Dis.

[30] Burri PH, McDonald JA (1997). Structural aspects of prenatal and postnatal development and growth of the lung. Lung growth and development. 100.

[31] Ochs M, Nyengaard JR, Jung A, Knudsen L, Voigt M, Wahlers T (2004). The number of alveoli in the human lung. Am J Respir Crit Care Med.

[32] Pozarska A, Rodriguez-Castillo JA, Surate Solaligue DE, Ntokou A, Rath P, Mizikova I (2017). Stereological monitoring of mouse lung alveolarization from the early postnatal period to adulthood. Am J Physiol Lung Cell Mol Physiol..

[33] Ten Have-Opbroek AA (1981). The development of the lung in mammals: an analysis of concepts and findings. Am J Anat.

[34] Burri PH (1984). Fetal and postnatal development of the lung. Annu Rev Physiol.

[35] Burri PH (2006). Structural aspects of postnatal lung development - alveolar formation and growth. Biol Neonate.

[36] Mund SI, Stampanoni M, Schittny JC (2008). Developmental alveolarization of the mouse lung. Dev Dyn.

[37] Zeltner TB, Caduff JH, Gehr P, Pfenninger J, Burri PH (1987). The postnatal development and growth of the human lung. I Morphometry Respir Physiol.

[38] Hislop AA (2002). Airway and blood vessel interaction during lung development. J Anat.

[39] Pringle KC (1986). Human fetal lung development and related animal models. Clin Obstet Gynecol.

[40] Zoetis T, Hurtt ME (2003). Species comparison of lung development. Birth Defects Res B Dev Reprod Toxicol.

[41] Langston C, Kida K, Reed M, Thurlbeck WM (1984). Human lung growth in late gestation and in the neonate. Am Rev Respir Dis.

[42] Thurlbeck WM (1975). Postnatal growth and development of the lung. Am Rev Respir Dis.

[43] Amy RW, Bowes D, Burri PH, Haines J, Thurlbeck WM (1977). Postnatal growth of the mouse lung. J Anat.

[44] Hislop AA, Wigglesworth JS, Desai R (1986). Alveolar development in the human fetus and infant. Early Hum Dev.

[45] Ardini-Poleske ME, Clark RF, Ansong C, Carson JP, Corley RA, Deutsch GH (2017). LungMAP: the molecular atlas of lung development program. Am J Physiol Lung Cell Mol Physiol.

[46] Krzyanowski MC, Levy J, Paige GP, Gaddis NC, Clark RF. Using semantic web technologies to power LungMAP, a molecular data repository. Proceeding SBD '17 Proceedings of The International Workshop on Semantic Big Data Article No 8 2017; Chicago, IL. New York ACM; 2017.

[47] Bandyopadhyay G, Huyck HL, Misra RS, Bhattacharya S, Wang Q, Mereness J (2018). Dissociation, cellular isolation, and initial molecular characterization of neonatal and pediatric human lung tissues. Am J Physiol Lung Cell Mol Physiol.

[48] Moghieb A, Clair G, Mitchell HD, Kitzmiller J, Zink EM, Kim YM (2018). Time-resolved proteome profiling of normal lung development. Am J Physiol Lung Cell Mol Physiol.

[49] Guo M, Du Y, Gokey JJ, Ray S, Bell SM, Adam M (2019). Single cell RNA analysis identifies cellular heterogeneity and adaptive responses of the lung at birth. Nat Commun.

[50] Ljungberg MC, Sadi M, Wang Y, Aronow BJ, Xu Y, Kao RJ (2019). Spatial distribution of marker gene activity in the mouse lung during alveolarization. Data Brief.

[51] Endale M, Ahlfeld S, Bao E, Chen X, Green J, Bess Z (2017). Temporal, spatial, and phenotypical changes of PDGFRalpha expressing fibroblasts during late lung development. Dev Biol.

[52] Musen MA, Protege T (2015). The Protege project: A look Back and a look forward. AI Matters.

[53] Noy NF, Crubezy M, Fergerson RW, Knublauch H, Tu SW, Vendetti J, et al. Protege-2000: an open-source ontology-development and knowledge-acquisition environment. AMIA Annu Symp Proc. 2003;953. PMC1480139 14728458

[54] Hayamizu TF, Wicks MN, Davidson DR, Burger A, Ringwald M, Baldock RA (2013). EMAP/EMAPA ontology of mouse developmental anatomy: 2013 update. J Biomed Semantics.

[55] Hayamizu TF, Baldock RA, Ringwald M (2015). Mouse anatomy ontologies: enhancements and tools for exploring and integrating biomedical data. Mamm Genome.

[56] Hayamizu TF, Mangan M, Corradi JP, Kadin JA, Ringwald M (2005). The adult mouse anatomical dictionary: a tool for annotating and integrating data. Genome Biol.

[57] Rosse C, Mejino JL (2003). A reference ontology for biomedical informatics: the foundational model of anatomy. J Biomed Inform.

[58] Mungall CJ, Torniai C, Gkoutos GV, Lewis SE, Haendel MA (2012). Uberon, an integrative multi-species anatomy ontology. Genome Biol.

[59] Diehl AD, Meehan TF, Bradford YM, Brush MH, Dahdul WM, Dougall DS (2016). The cell ontology 2016: enhanced content, modularization, and ontology interoperability. J Biomed Semantics.

[60] Meehan TF, Masci AM, Abdulla A, Cowell LG, Blake JA, Mungall CJ (2011). Logical development of the cell ontology. BMC Bioinformatics.

[61] Jeffery PK, Gaillard D, Moret S (1992). Human airway secretory cells during development and in mature airway epithelium. Eur Respir J.

[62] Jeffrey PK (1998). The development of large and small airways. Am J Respir Crit Care Med.

[63] LeCras TD, Rabinovitch M, Jobe AH, Whitsett JA, Abman SH (2016). Pulomonary vascular development. Fetal and neonatal lung development: clinical correlates and Technologies for the Future.

[64] Sparrow MP, Lamb JP (2003). Ontogeny of airway smooth muscle: structure, innervation, myogenesis and function in the fetal lung. Respir Physiol Neurobiol.

[65] Sparrow MP, Weichselbaum M, McCray PB (1999). Development of the innervation and airway smooth muscle in human fetal lung. Am J Respir Cell Mol Biol.

[66] Tollet J, Everett AW, Sparrow MP (2001). Spatial and temporal distribution of nerves, ganglia, and smooth muscle during the early pseudoglandular stage of fetal mouse lung development. Dev Dyn.

[67] Albertine KH, Mason RJ, Broaddus VC, Martin TE, King TE, Schraufnagel DE, Murray JF (2010). Anatomy of The Lungs. Murry & Nadel’s Textbook of Respiratory Medicine.

[68] Breeze RG, Wheeldon EB (1977). The cells of the pulmonary airways. Am Rev Respir Dis.

[69] Crapo JD, Barry BE, Gehr P, Bachofen M, Weibel ER (1982). Cell number and cell characteristics of the normal human lung. Am Rev Respir Dis.

[70] Franks TJ, Colby TV, Travis WD, Tuder RM, Reynolds HY, Brody AR (2008). Resident cellular components of the human lung: current knowledge and goals for research on cell phenotyping and function. Proc Am Thorac Soc.

[71] Sorokin S, Weiss L (1988). The respiratory system. Cell and tissue biology, A textbook of histology.

[72] Ding J, Aronow BJ, Kaminski N, Kitzmiller J, Whitsett JA, Bar-Joseph Z. Reconstructing differentiation networks and their regulation from time series single-cell expression data. Genome Res. 2018;28:383–95. 10.1101/gr.225979.117 PMC5848617 29317474

[73] Guo M, Bao EL, Wagner M, Whitsett JA, Xu Y (2017). SLICE: determining cell differentiation and lineage based on single cell entropy. Nucleic Acids Res.

[74] Guo M, Wang H, Potter SS, Whitsett JA, Xu Y (2015). SINCERA: A pipeline for single-cell RNA-Seq profiling analysis. PLoS Comput Biol.

[75] Trapnell C (2015). Defining cell types and states with single-cell genomics. Genome Res.

[76] Bard JB (2005). Anatomics: the intersection of anatomy and bioinformatics. J Anat.

[77] Smith B, Ceusters W, Klagges B, Kohler J, Kumar A, Lomax J (2005). Relations in biomedical ontologies. Genome Biol.

[78] Musen MA, Noy NF, Shah NH, Whetzel PL, Chute CG, Story MA (2012). The National Center for biomedical ontology. J Am Med Inform Assoc.

[79] Whetzel PL, Noy NF, Shah NH, Alexander PR, Nyulas C, Tudorache T (2011). BioPortal: enhanced functionality via new web services from the National Center for biomedical ontology to access and use ontologies in software applications. Nucleic Acids Res.

[80] Bard J (2012). A new ontology (structured hierarchy) of human developmental anatomy for the first 7 weeks (Carnegie stages 1-20). J Anat.

[81] Weibel ER, Gomez DM. Architecture of the human lung. Use of quantitative methods establishes fundamental relations between size and number of lung structures. Science. 1962;137:577–85. 10.1126/science.137.3530.577 14005590

[82] Weibel ER (1963). Principles and methods for the morphometric study of the lung and other organs. Lab Investig.

[83] Bucher U, Reid L (1961). Development of the intrasegmental bronchial tree: the pattern of branching and development of cartilage at various stages of intra-uterine life. Thorax.

[84] Irvin CG, Bates JHT. Measuring the lung function in the mouse: the challenge of size. Respir Res. 2003;4:4. 10.1186/rr199 PMC184039 12783622

[85] Blanc PCK, Pouchin P, Azaïs JM, Blanchon L, Gallot D, Sapin V. A role for mesenchyme dynamics in mouse lung branching morphogenesis. PLoS One. 2012;7:(7):e41643. 10.1371/journal.pone.0041643 PMC3402475 22844507

[86] Metzger RJ, Klein OD, Martin GR, Krasnow MA (2008). The branching programme of mouse lung development. Nature.

[87] Reynolds SD, Pinkerton KE, A.T., Parent RA (2015). M. Epithelial cells of trachea and bronchi. Comparative biology of the Normal lung.

[88] Plopper CG, D.M. H., Parent RA (2015). Epithelial cells of the bronchiole. Comparative biology of the Normal lung.

[89] Boers JE, Ambergen AW, Thunnissen FB (1999). Number and proliferation of clara cells in normal human airway epithelium. Am J Respir Crit Care Med.

[90] Plopper CG, Hyde DM (2008). The non-human primate as a model for studying COPD and asthma. Pulm Pharmacol Ther.

[91] Berend N, Rynell AC, Ward HE (1991). Structure of a human pulmonary acinus. Thorax.

[92] Du Y, Guo M, Whitsett JA, Xu Y (2015). 'LungGENS': a web-based tool for mapping single-cell gene expression in the developing lung. Thorax.

[93] Du Y, Kitzmiller JA, Sridharan A, Perl AK, Bridges JP, Misra RS (2017). Lung gene expression analysis (LGEA): an integrative web portal for comprehensive gene expression data analysis in lung development. Thorax.

[94] Chen J, Bardes EE, Aronow BJ, Jegga AG (2009). ToppGene suite for gene list enrichment analysis and candidate gene prioritization. Nucleic Acids Res.

[95] Kaimal V, Bardes EE, Tabar SC, Jegga AG, Aronow BJ (2010). ToppCluster: a multiple gene list feature analyzer for comparative enrichment clustering and network-based dissection of biological systems. Nucleic Acids Res.

[96] Desai TJ, Brownfield DG, Krasnow MA (2014). Alveolar progenitor and stem cells in lung development, renewal and cancer. Nature.

[97] Jain R, Barkauskas CE, Takeda N, Bowie EJ, Aghajanian H, Wang Q (2015). Plasticity of Hopx(+) type I alveolar cells to regenerate type II cells in the lung. Nat Commun.

[98] Treutlein B, Brownfield DG, Wu AR, Neff NF, Mantalas GL, Espinoza FH (2014). Reconstructing lineage hierarchies of the distal lung epithelium using single-cell RNA-seq. Nature.

