# Supplementary material for: Comprehensive anatomic ontologies for lung development: A comparison of alveolar formation and maturation within mouse and human lung
Source: J Biomed Semantics. 2019 Oct 24;10:18. doi: 10.1186/s13326-019-0209-1 (PMC6814058; doi:10.1186/s13326-019-0209-1)
Supplement: Supplementary file 1 — Additional file 1. OWL file for the class left lung alveolar parenchyma with its annotation properties and relationships. [file 13326_2019_209_MOESM1_ESM.docx]

**Additional File 1.** OWL file for the class *left lung alveolar parenchyma* with its annotation properties and relationships.

<owl:Class rdf:about="http://www.lungmap.net/ontologies/mouse_anatomy#LMMA_00339">

<rdfs:label>secondary alveolar septum</rdfs:label>

<rdfs:subClassOf>

<owl:Restriction>

<owl:onProperty rdf:resource="http://www.lungmap.net/ontologies/mouse_anatomy/part_of"/>

<owl:someValuesFrom rdf:resource="http://www.lungmap.net/ontologies/mouse_anatomy#LMMA_00002"/>

</owl:Restriction>

</rdfs:subClassOf>

<oboInOwl:hasDbXref rdf:datatype="http://www.w3.org/2001/XMLSchema#string">UBERON:0005315 </oboInOwl:hasDbXref>

<breath_database:Theiler_Stage rdf:resource="http://www.lungmap.net/ontologies/mouse_anatomy#LMMA_00375"/>

<breath_database:develops_from rdf:resource="http://www.lungmap.net/ontologies/mouse_anatomy#LMMA_00071"/>

<breath_database:develops_from rdf:resource="http://www.lungmap.net/ontologies/mouse_anatomy#LMMA_00279"/>

<breath_database:display>true</breath_database:display>

<breath_database:display_order rdf:datatype="http://www.w3.org/2001/XMLSchema#integer">3</breath_database:display_order>

<breath_database:evidence>published</breath_database:evidence>

<breath_database:has_domain rdf:resource="http://www.lungmap.net/ontologies/database#anatomy"/>

<breath_database:in_organism rdf:resource="http://www.lungmap.net/ontologies/data#taxid_10090"/>

<breath_database:in_stage rdf:resource="http://www.lungmap.net/ontologies/mouse_anatomy#LMMA_00266"/>

<breath_database:in_stage rdf:resource="http://www.lungmap.net/ontologies/mouse_anatomy#LMMA_00268"/>

<breath_database:in_stage_range rdf:resource="http://www.lungmap.net/ontologies/mouse_anatomy#LMMA_00670"/>

<rdfs:comment>primary alveolar septum gives rise to the alveolar septal crest, which develops into secondary alveolar septum</rdfs:comment>

</owl:Class>

______________________________________________________________________________
