# Supplementary material for: Comprehensive anatomic ontologies for lung development: A comparison of alveolar formation and maturation within mouse and human lung
Source: J Biomed Semantics. 2019 Oct 24;10:18. doi: 10.1186/s13326-019-0209-1 (PMC6814058; doi:10.1186/s13326-019-0209-1)
Supplement: Supplementary file 2 — Additional file 2.. Specific differences in lung structure between human and mouse. [file 13326_2019_209_MOESM2_ESM.docx]

| Additional File 2. Specific differences in lung structure between human and mouse. | | |  |
| --- | --- | --- | --- |
| Feature | Human | Mouse | |
| Lobes | 3 Right/2 Left | 4 Right/1 Left | |
| Cartilaginous airway rings | Trachea to intrapulmonary segmental bronchi | Trachea to extrapulmonary lobar bronchi | |
| Submucosal glands | Trachea, main, lobar and segmental bronchi | Trachea | |
| Bronchopulmonary segments | 10 Right/8-10 Left | No | |
| Pulmonary lobules | Yes | No | |
| Terminal airways | Respiratory bronchiole, alveolar duct | Terminal bronchiole, bronchioalveolar duct junction, alveolar duct | |
| Interlobular septa | Yes | No | |
| Pulmonary veins | Smooth muscle wall/  located in interlobular septa | Cardiac and smooth muscle wall/located in alveolar parenchyma | |
| Visceral pleura | Thick connective tissue compartment with superficial lymphatic network | Single layer of mesothelial cells overlying thin connective tissue layer | |
