# Supplementary figures and images for: Comprehensive anatomic ontologies for lung development: A comparison of alveolar formation and maturation within mouse and human lung
Source: J Biomed Semantics. 2019 Oct 24;10:18. doi: 10.1186/s13326-019-0209-1 (PMC6814058; doi:10.1186/s13326-019-0209-1)

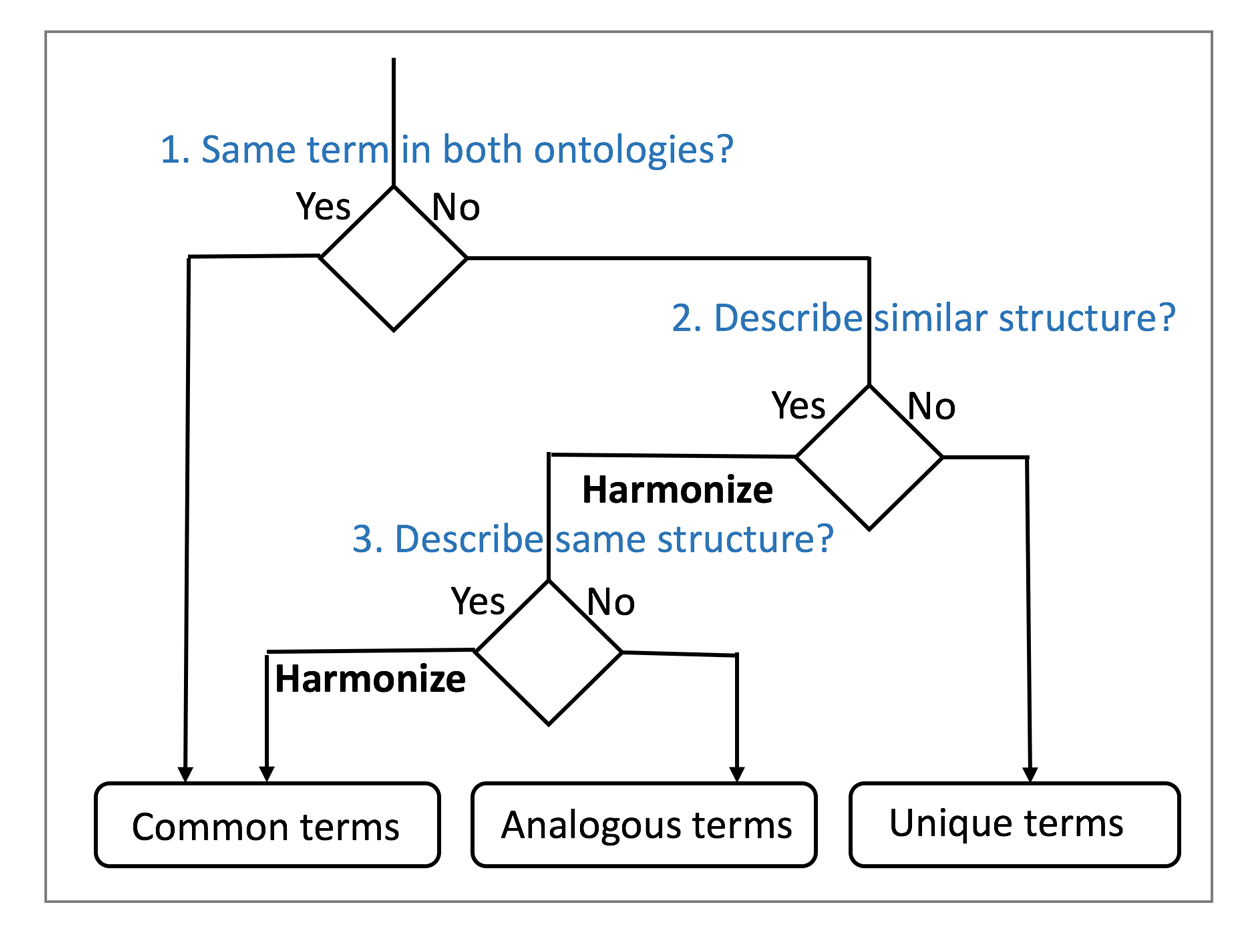

Supplement: Supplementary file 4 — Additional file 4.. Harmonization process comparing anatomic structures and terms in human and mouse ontologies. [file 13326_2019_209_MOESM4_ESM.tif]
